# Pregnant Women's Experiences With a Collaborative Midwife‐Dietitian Empowerment Programme to Improve Diet Quality

**DOI:** 10.1111/jhn.70027

**Published:** 2025-02-11

**Authors:** Renske M. van Lonkhuijzen, Suzanne Prins, Fleur van Loghem, Jeanne H. M. de Vries, Annemarie Wagemakers

**Affiliations:** ^1^ Health and Society, Department of Social Sciences Wageningen University & Research Wageningen the Netherlands; ^2^ Human Nutrition & Health, Department of Agrotechnology and Food Sciences Wageningen University & Research Wageningen the Netherlands; ^3^ Municipal Health Services Twente Enschede the Netherlands; ^4^ The Netherlands Nutrition Centre The Hague the Netherlands

**Keywords:** diet quality, dietitian, empowerment, interviews, midwife, pregnancy

## Abstract

**Background:**

Pregnancy is a crucial period prompting increased intentions for lifestyle changes. Suboptimal diet quality during pregnancy can cause adverse health outcomes for both mother and child. The Power 4 a Healthy Pregnancy (P4HP) programme aims to improve the diet quality of pregnant women through empowerment, by providing four additional consultations to discuss nutrition with a midwife and dietitian. This research aimed to study the experiences of pregnant women engaged in the P4HP programme.

**Methods:**

A qualitative study using in‐depth interviews was conducted. Participants were recruited through purposive sampling from women who completed the P4HP programme. Semi‐structured interviews were conducted using time‐lining as an explorative tool. Interview transcripts underwent thematic analysis following Braun and Clarke's six‐phase process, combining inductive and deductive coding approaches.

**Results:**

Twenty‐two interviews were conducted with women from eight midwifery practices. Four main themes emerged: (1) women report various dietary improvements, influenced by diverse factors, (2) most pregnant women evaluate the P4HP programme positively, (3) the dietitian plays a key role in empowering pregnant women towards healthy dietary intakes and (4) midwives support pregnant women in maintaining dietary improvements. Participants viewed the P4HP consultations favourably, which facilitated empowerment through personalized guidance, reassurance, and increased awareness and confidence levels. Motivated by their desire to ensure optimal nutrition for their babies, women made multiple improvements in their diet quality. While the guidance of the midwife served as a motivational factor to sustain these changes, it was the personalized nutritional guidance provided by the dietitian that women found instrumental in achieving actual dietary changes.

**Conclusion:**

Our outcomes emphasize the importance of integrating dietitian consultations in standard antenatal care to promote enhancements in the diet quality of pregnant women.

## Summary

- Adhering to dietary guidelines is crucial during pregnancy for the well‐being of both mother and child, but multifaceted challenges hinder compliance.
- Pregnant women perceived the Power 4 a Healthy Pregnancy consultations favourably, as they raised awareness, reassurance and confirmation regarding the quality of their dietary intake, thereby increasing their confidence.
- The collaborative engagements of dietitians and midwives play a vital and promising role in the empowerment of pregnant women to improve their dietary behaviours.

## 1 Introduction

Maintaining a healthy diet is essential for everyone, but is especially crucial for pregnant women to ensure the health of both mother and child [1, 2]. Because pregnant women are aware of the importance of the healthy development of their baby, they tend to be more willing to change dietary habits and seek out information regarding antenatal diet [3, 4, 5, 6]. In a study among 343 Dutch women, 56% of participants reported increased lifestyle change intentions because of their pregnancy [7]. Pregnancy can therefore be seen as a teachable moment and vital transition period during which women are more receptive to improving their dietary choices than compared to other life stages.

Poor diet quality during pregnancy increases the mother's risk of gestational diabetes, excessive weight gain and pre‐eclampsia, as well as increasing the likelihood of low birth weight, premature childbirth or future adverse health outcomes for the child, such as chronic diseases [8, 9, 10, 11]. According to the World Health Organization [12], these guidelines include taking adequate energy, protein, vitamins and minerals, obtained through the consumption of a variety of foods, including green and orange vegetables, meat, fish, beans, nuts, whole grains and fruit. In the Netherlands, specific dietary guidelines tailored for pregnant women have been established [13]. These guidelines for pregnant women recommend taking adequate amounts of vitamin D, folic acid, iodine, iron and calcium while advising them to avoid certain food groups, such as alcohol and liver products, for the proper development of the foetus. Adherence to an adequate diet during and before pregnancy is crucial to benefit the health of both the mother and baby, as well as the child's well‐being during later life.

Pregnant women in general, and particularly those with low socioeconomic status (SES), struggle to adhere to the dietary guidelines [14, 15]. Women face several physiological challenges during pregnancy that could hinder healthy eating, such as nausea, cravings, tiredness and food aversions [16, 17]. In addition, pregnant women can experience multiple barriers: social barriers (e.g., lack of support, peer beliefs); psychological barriers (e.g., habits, emotional stress); cognitive barriers (e.g., lack of knowledge); emotional barriers (e.g., preference); interpersonal barriers (e.g., time, habits) and environmental challenges (e.g., availability of healthy foods) [5, 16, 17, 18]. Considering these multifaceted challenges, supporting pregnant women in adhering to dietary guidelines is important for maternal and foetal health.

In the Netherlands, midwives are the first informant for pregnant women to discuss nutrition. They perceive themselves as crucial in providing nutritional advice and guidance [19, 20], even though nutrition communication is not explicitly mentioned in the counselling recommendations of Dutch midwives [21]. However, due to constraints including insufficient resources, knowledge and time, midwives often struggle to provide adequate nutritional advice, typically limiting recommendations to general dietary guidelines [5, 22, 23]. Currently, often only pregnant women with medical issues such as excessive weight gain are routinely referred to a dietitian. However, research shows that low SES pregnant women would appreciate support in achieving a good diet quality, regardless of medical issues [24, 25]. Therefore, dietitians could bridge this gap in prenatal care, offering their expertise in personalized nutritional guidance [20, 25]. Previous research has shown that midwives consider collaboration with other health professionals, especially dietitians, as a good strategy to optimize the provision of nutritional advice [19]. Nevertheless, collaboration between midwives and dietitians is currently limited in antenatal care [20].

The Power 4 a Healthy Pregnancy (P4HP) programme has been developed to improve the diet quality of pregnant women through empowerment [5, 26, 27]. To develop the P4HP programme, an iterative research process was undertaken to identify strategies to improve the diet quality of pregnant women. Development of the P4HP programme involved literature reviews, expert consultations, focus groups and interviews with pregnant women from low SES, midwives and dietitians. In the final developmental stage, three co‐creation sessions with stakeholders further improved P4HP with input from various prenatal care providers. As a result, the P4HP programme is a flexibly adaptable programme in which midwives, dietitians and pregnant women collaborate to improve the diet quality of pregnant women through empowerment. Although initially designed for low‐SES pregnant women, the P4HP programme is implemented among women of all socioeconomic backgrounds [27]. The programme facilitates four additional nutrition consultations during pregnancy with three conducted by midwives and one by dietitians. Midwives and dietitians were equipped with a comprehensive guide for the implementation of the P4HP programme (Supporting Information S1: File 1). The P4HP programme employs an empowering approach through Motivational Interviewing (MI). MI is a counselling method that recognizes women as experts in their own lives and is used to increase motivation, self‐efficacy and personal control among pregnant women to eat healthily [28, 29]. This approach aims to support women in making autonomous decisions about their dietary choices during pregnancy, rather than prescribing strict dietary rules. During P4HP consultations, midwives and dietitians used MI techniques to explore women's motivations and barriers regarding healthy eating, assisting them in identifying personally meaningful dietary goals and strategies. A visual conversation tool is employed, enabling pregnant women to mark the food groups they want to discuss during consultations, thereby promoting commitment to dietary improvements [30]. Emphasizing a women‐centred approach, the P4HP programme supports individual needs and autonomy, stimulating pregnant women to voice personal objectives [27].

The P4HP programme has been implemented and evaluated using a non‐blinded clustered randomized trial (C‐RCT) across 16 Dutch midwifery practices including 342 pregnant women. Total diet quality significantly improved in the intervention group, which will be reported elsewhere [31]. It is unknown how pregnant women experience and evaluate the P4HP programme, and why. Studies on empowerment‐based programmes aimed at promoting healthy nutrition during pregnancy are scarce [32, 33]. Currently, similar programmes, utilizing empowerment to improve the diet quality of pregnant women, do not exist. The added value of this research lies in its novel focus on evaluating the experiences of pregnant women participating in an empowerment programme aimed at improving diet quality. The objective of this qualitative study is therefore to examine (1) reported changes in diet quality among pregnant women and the factors influencing these changes and (2) pregnant women's perceptions of the P4HP programme.

## 2 Methods

### 2.1 Study Design

This descriptive qualitative study aimed to explore the experiences of pregnant women who followed the P4HP programme [27, 34, 35]. Participant recruitment and data collection were performed from October to December 2022. Data collection involved in‐depth interviews conducted by two researchers. Participation in this evaluation study was voluntary and participants were free to withdraw at any time without consequences or the need to disclose their reasons. Participants provided written informed consent before each interview.

### 2.2 Participant Recruitment

**Figure 1:**
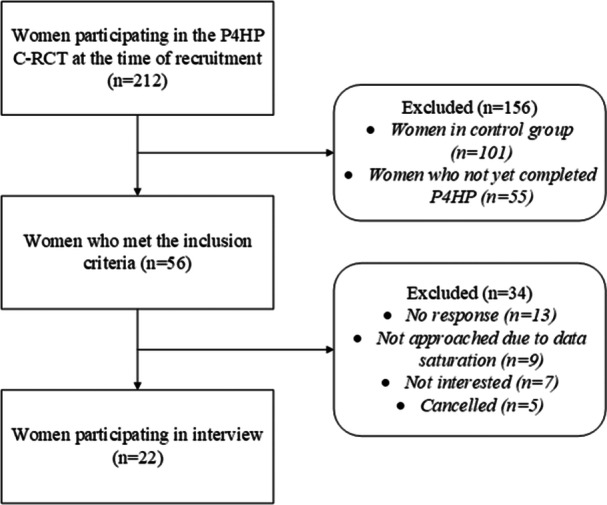
Flowchart of the recruitment procedure of the participants.

Pregnant women who were participating in the P4HP C‐RCT were invited to take part in this follow‐up study [27, 31]. At the time of recruitment for our study, 212 women were enrolled in the P4HP C‐RCT. The C‐RCT inclusion criteria required women to be in their first trimester of pregnancy, over 18 years of age, fluent in Dutch and following a typical Dutch dietary pattern (characterized by one hot meal per day). Women were excluded if they declined to provide informed consent, had severe chronic illnesses (e.g., cancer) or conditions that could affect diet quality [27, 31]. To be included in our study, participants needed to have fully completed the intervention part of the P4HP programme and given informed consent for follow‐up research (Figure 1). We used a purposive sampling method, initially contacting potential participants by phone and sending follow‐up emails if there was no response. Participant recruitment occurred in two phases. First, we recruited all women who met the inclusion criteria. Then, we took a more targeted approach, seeking women with specific characteristics that were not fully represented in the initial sample. Specifically, we reached out to women from two midwifery practices where they received two consultations with a dietitian as part of the P4HP programme, rather than one [31], to delve deeper into their experiences.

### 2.3 Data Collection

The interviews were conducted by two female researchers (F.L. and S.P.), who were MSc students in Health & Society at the time and had undertaken training in qualitative research methodologies. Interviews were audio‐recorded using a dictation device and conducted with only the participant and researchers present, who had no prior relationship with each other. F.L. and S.P. alternated as the lead and supporting interviewer. To ensure a safe and supportive environment, the interviews were held at the woman's house. Before each interview, participants were informed about the researchers’ affiliations and the full purpose of the research. Before starting, the researchers had a brief informal chat with each woman and reiterated that it was acceptable to avoid discussing uncomfortable topics. At the end of each interview, the interviewee received a children's book (~€15) as a token of appreciation.

A semi‐structured interview guide was developed after extensive review by all authors (Supporting Information S1: File 2), allowing for open conversation, and enabling researchers to ask probing questions and explore the topics in depth. The interviews began with open‐ended questions about the woman's pregnancy experience, followed by questions on significant events during pregnancy, experience with the P4HP programme, perceived diet quality and empowerment. Questions on diet quality were inspired by the inner circle of the wheel‐shaped framework by Super and Wagemakers [5], describing five perspectives on diet quality during pregnancy: (1) health‐promoting foods and products, (2) challenges in healthy eating, (3) risky products, (4) strategies for healthy eating and (5) motivational aspects. For example, for strategies and challenges, we asked: ‘Did you find it difficult or easy to adhere to this eating pattern, and why?’

To prepare for the interviews, the interviewers (F.L. and S.P.) read documents about the interviewees to familiarize themselves with the participants. The documents included information, documented by midwives and dietitians during the P4HP consultations, on the participant's goals, motivations, barriers and strategies. Interview questions were not altered based on this information. The data in these documents corresponded with the findings of the interviews in 21 of the 22 cases. In a single case, the document stated that the woman struggled with healthy eating while, in contrast, the woman said during the interview that she did not struggle and that her pregnancy was actually a motivator for eating healthier.

During the interviews, an explorative timelining tool was used whenever possible to enhance communication, improve data validity and quality, and facilitate the relationship between the researcher and the participant [36]. We drew two parallel timelines on an A3‐sized sheet, one for the pregnancy period and the other for the P4HP programme, including time points of the four consultations. The tool served as a visual aid, combined with questions about the woman's pregnancy and consultations, to help her identify ‘trigger points’ that led to changes in diet quality during pregnancy [37]. To actively engage the woman, we asked her to write the trigger points on the timeline herself, guiding the interview to explain her experience with the P4HP programme and her pregnancy. Field notes were taken during the timelining process and were incorporated into the analysis. Timelining was used in 14 of the 22 interviews. Timelining was not used in case of practical constraints (such as holding a baby or being in an unsuitable environment) or when assessing the tool would not be beneficial for the specific interview situation.

Throughout the data collection period, all authors regularly discussed emerging themes and patterns. After 17 interviews, new data primarily reinforced existing themes rather than generating new insights related to dietary changes, influencing factors and programme experiences. To confirm this, we conducted five additional interviews, bringing the total to 22 interviews [38].

### 2.4 Data Analysis

The audio recordings of the interviews were transcribed using intelligent verbatim transcription, leaving out filler words (i.e., ‘hmm’ and ‘uh’). Transcripts were then analysed in ATLAS.ti, version (ATLAS.ti Scientific Software Development GmbH, Berlin, Germany) using a hybrid coding approach, combining both deductive codes based on existing theoretical frameworks with inductive codes emerging directly from the interview data [39]. This approach allowed for systematic analysis while remaining open to new insights. All interviews were conducted and initially analysed in Dutch. Relevant quotes were translated to English by F.L. and S.P. and verified by R.M.L. and A.W. to ensure accuracy. For the thematic analysis, we followed Braun and Clarke's six‐phase process [40, 41, 42, 43] to identify patterns within the data. The transcription and coding processes were done by two researchers (F.L. and S.P.) alternately. Initially, the interviews were transcribed to familiarize the researchers with the data. Subsequently, we explored the data by thoroughly reading and rereading the data to identify codes. Using a combination of inductive and deductive coding [39, 44], we generated initial themes. Deductive codes were based on the wheel‐shaped framework by Super and Wagemakers [5], which included categories, such as healthy foods products, strategies and challenges to healthy eating, and the social environment. Next, potential themes were repeatedly reviewed and discussed among authors (R.M.L., S.P., F.L. and A.W.), with any uncertainties regarding codes or themes resolved through collective consensus. Finally, we present our findings and interpretation of the themes and analysis in the Results section.

## 3 Results

A total of 22 women participated in this study, with ages ranging from 25 to 40 years (mean age 31 years). Participants were recruited from eight distinct midwifery practices (A–H) (Table 1). At the time of the interviews, 8 women were pregnant and 14 were postpartum. The interviews lasted between 26 and 61 min, with an average duration of 41 min (see Table 1 for detailed participant characteristics). Analysis of the interview data revealed four main themes: (1) women report various dietary improvements, influenced by diverse factors, (2) most pregnant women evaluate the P4HP programme positively, (3) the dietitian plays a key role in empowering pregnant women towards healthy dietary intakes and (4) midwives support pregnant women in maintaining dietary improvements.

**Table 1:** Participants characteristics of 22 pregnant and postpartum women participating in the qualitative evaluation of Power 4 a Healthy Pregnancy programme.

Participant	Age	Highest attained education level	Personal monthly net income (€)	Household monthly net income (€)	Average working hours a week	Week of pregnancy (P)/weeks postpartum (PP)	Number of children	Midwifery practice	Time of interview (minutes)
P1	28	University level education	2000–2500	5000+	28	36 P	1	A	41
P2	29	Higher vocational training	2000–2500	5000+	32	38 P	1	A	61
P3	37	University level education	2000–2500	5000+	40	4 PP	1	B	35
P4	36	Secondary vocational training	Unknown	Unknown	12	36 P	2	C	37
P5	25	Secondary education	2500+	4500–5000	32	5 PP	2	B	40
P6	34	Secondary education	< 500	3500–4000	0	5 PP	2	D	26
P7	40	Secondary education	2000–2500	5000+	36	38 P	1	D	44
P8	31	University level education	1000–1500	3000–35,000	28	3 PP	2	B	38
P9	34	University level education	2500+	5000+	32	4 PP	1	B	44
P10	26	Higher vocational training	1500–2000	4000–4500	36	6 PP	1	B	38
P11	32	University level education	2500+	5000+	36	12 PP	1	B	43
P12	29	Higher vocational training	2500+	5000+	34	16 PP	1	B	44
P13	40	University level education	2500+	5000+	32	12 PP	1	B	58
P14	36	Secondary vocational training	1000–1500	Unknown	12	4 PP	4	E	44
P15	31	University level education	2500+	5000+	32	36 P	1	F	37
P16	31	Higher vocational training	2000–2500	5000+	24	5 PP	2	G	41
P17	31	Higher vocational training	2000–2500	5000+	36	38 P	1	D	53
P18	29	Higher vocational training	2000–2500	5000+	40	9 PP	1	D	51
P19	26	Secondary vocational training	< 500	2000–2500	0	7 PP	6	C	38
P20	28	Higher vocational training	1500–2000	4000–4500	24	36 P	2	E	33
P21	25	Secondary vocational training	1000–1500	3000–3500	20	37 P	2	E	32
P22	29	Higher vocational training	2500+	4500–5000	30	4 PP	2	H	32

### 3.1 Theme 1: Women Report Various Dietary Improvements, Influenced by Diverse Factors

During pregnancy, all 22 women in this study made changes to their diet. On average, women mentioned changing the intake of seven types of foods during pregnancy with a range from 3 to 10 changes per woman. Table 2 presents the primary reported changes in diet quality, explains the change and presents examples of quotes regarding dietary changes. Making dietary improvements was influenced by physical well‐being, reasons for changing diet quality, challenges, feelings of responsibility and the postpartum period.

**Table 2:** Reported primary improvements in diet during pregnancy by participating women (*n* = 22), with explanation and example quotes.

Food product	More or less consumption	Explanation	Example quote
Dairy products	More (*n* = 15)	Women frequently increased their consumption of milk (*n* = 11) and yoghurt (*n* = 11). To provide women with strategies to obtain adequate calcium and protein intake, the dietitian recommended women to consume more dairy foods, for example, by always drinking a glass of (butter)milk or yoghurt drink with a meal, consume a dairy food during a snack moment or to switch to supplements. Also, some midwives recommended taking supplements when women found it difficult to obtain sufficient calcium through their diet.	PW4: ‘I mentioned that I eat more yoghurt now and she [the dietitian] said I can do that much more. Just like I get a dip around 4 PM, I can also take yoghurt with some muesli for example. That kind of things, as a tip that you can also do that at other times of the day’.
Fish	More (*n* = 8)	The dietitian frequently emphasized the importance of eating more fish during pregnancy, primarily to obtain omega‐3 fats. Almost every interview included a remark on fish (*n* = 18) and many women started eating more fish or tried to during their pregnancy (*n* = 8). Women experienced challenges regarding fish intake because it is expensive, they don't usually purchase fish, many types of fish are not allowed during pregnancy, or women or partners disliked fish. To provide women with strategies to obtain sufficient omega‐3 fats, dietitians advised buying cheaper (frozen) fish from the supermarket, cooking it thoroughly, or choosing alternatives such as nuts or supplements.	PW5: ‘She [the dietitian] was pleased with what I said about it being difficult to know which fish is safe to eat. It's also quite expensive to go to the fishmonger and get a fresh piece of fatty fish, so I didn't do that often. She said: well then don't buy fatty fish often, but just white fish from the freezer in the supermarket. I thought: oh, it doesn't have to be all or nothing. […] That's not optimal but it's at least something, so I started doing that’.
Supplements	More (*n* = 8)	In addition to taking nutrient supplements targeted for pregnancy, women started supplementing if they struggled with getting enough calcium or fish oil containing omega‐3 fats as a strategy to get sufficient micronutrients. This strategy was frequently recommended by the dietitian or midwife, but some women also initiated this themselves.	PW17: ‘[…] that [the recommended dairy intake] was a lot of glasses of milk, so then I decided to also take calcium supplements because I knew that I would never make it’.
Fruit & vegetables	More (*n* = 13)	Women frequently increased their fruit and vegetable intake during pregnancy (*n* = 13), with 10 women eating more fruit and nine eating more vegetables. Many women became aware of their diet quality through pregnancy and recognized the importance of having an adequate fruit and vegetables intake to meet their nutrient requirements. To provide women with strategies to eat enough fruit and vegetables, the dietitian recommended blending fruit and vegetables into smoothies, eating them as a snack after or between meals, or adding vegetables to sandwich fillings. Other strategies of women were to curb sweet cravings with fruit or incorporate vegetable snacks in their meals	PW18: ‘I really craved something sweet, and with fruit, I could satisfy that […]. So I started eating a lot of strawberries and apples, and really sweet fruit like mango’.
Water	More (*n* = 9)	Many women experienced drinking water as a challenge. Before pregnancy, nine women struggled with this, but during pregnancy women realized the importance of drinking enough water for themselves and for the baby and therefore started doing or attempting to do more. Women faced challenges such as not feeling thirsty or finding drinking water boring. To provide women with strategies to drink sufficient water, dietitians recommended adding a flavour to make water taste better, setting an alarm, or keeping bottles of water in sight. A couple of women replaced soft drinks and coffee with water or tea, as recommended by the dietitian.	PW6: ‘I also ended up just filling up the fridge eventually with some more flavored water and things like that, because of the advice [of the dietitian]. Of course, water is pretty boring if you drink that every day’.
(Whole grain) bread	More (*n* = 8)	Many women were unaware that they required additional nutrition while pregnant. Eight women started eating more whole grain slices of bread, as recommended by the dietitian to feel longer satiated as their appetite increased, to improve nausea, or as a substitute for wheat products or (rice) crackers. Most women followed the dietitian's advice, but a few struggled because they disliked bread or did not eat it regularly. A dietitian recommended storing bread in the freezer as a strategy to make it easy to take bread. Some women had to get used to eating that much bread in a day since it was not a habit.	PW21: ‘[…] and she [the dietitian] said that I was allowed to eat more whole grain products and bread. Soon after the nausea is gone, you just eat ‘normally’ again. I think she said to have 3 or 5 slices of bread a day, which I thought was a lot, but apparently it's allowed’.
Unsalted unroasted nuts	More (*n* = 7)	The dietitian often recommended consuming more unsalted, unroasted nuts, as they are a nutrient‐dense food group. In the interviews, these were commonly referred to as ‘healthy nuts’. Almost a third of the women (*n* = 7) consumed more nuts as a replacement for unhealthy snacks or to feel more satiated. However, women frequently encountered challenges such as the fact that nuts are expensive, it is not a habit for them to grab them, they do not like them, or nuts are high in energy.	PW8: ‘Now I take a small container with me with some nuts and a few raisins to keep it somewhat healthy, and then it's also good because the nuts fill up quite well’.
Sugar	More (*n* = 11) or less (*n* = 4)	Eleven women increased the consumption of sugary food products or beverages (including fruit juices) during pregnancy, whereas four women consumed less sugar during pregnancy. Women limited sugar intake because of a sense of responsibility for the baby's health and fear of gaining excessive weight. However, many women admitted that they struggled with sweet cravings, mentioning chocolate in particular. Resisting temptations became increasingly challenging as women's appetites increased throughout pregnancy. To provide women with strategies to cope with temptations and cravings, the dietitian recommended eating more whole grain or dairy products during the day to feel more satiated, to replace sugary drinks with water or tea or to replace sugary food products with healthy snacks women like such as nuts, fruits, or dairy products.	I: ‘Was it a conscious choice to drink sodas with more sugar before pregnancy?’ PW1: ‘No, I was already drinking it, but more often. So since the onset of pregnancy, I have really started paying attention to limiting it to one glass a day.’ I: ‘And what was the reason for you doing that now and not before pregnancy?’ PW1: ‘I think there was a little fear that you will gain a lot of weight’.
Risky food products	Less (*n* = 11)	Half of the women explicitly reported consuming fewer unsafe foods during pregnancy, such as raw products, red meat, certain types of fish and coffee. While no explicit reasons were given for this reduction, the risks of certain food groups are well‐known among pregnant women. As consuming unsafe food products was a habit for women, they had to actively avoid them during pregnancy. Women found it challenging to give up these prohibited food groups and looked forward to consuming them again after pregnancy. Almost all women consulted *Zwangerhap* from The Netherlands Nutrition Centre, a mobile application to check whether food products are safe to eat during pregnancy, if they were uncertain whether particular foods were safe to consume during pregnancy. Most women found the app clear enough, but some discussed ambiguities with the dietitian such as how to handle certain types of cheeses. Besides, the dietitian recommended how to consume certain unsafe food products by heating them properly or looking for alternatives.	I: ‘If you had any doubts about the Zwangerhap app, did you discuss that with the dietitian?’ PW15: ‘Yes, I remember we talked about cheese as well, because it's not always clear which ones are okay and which ones aren't. For example, if you heat it, is it safe or not? These are the kinds of things you can't find on the internet very well […]. So it was great that I could ask her’.

The diet quality of pregnant women fluctuates during pregnancy, mainly influenced by their physical well‐being. Many women experience reduced appetite during the first trimester of their pregnancy, due to nausea, causing them to eat less. As most consultations with the dietitian took place at the end of the first trimester, many women received targeted advice on managing nutrition during nausea, such as consuming crackers or fruit. Later in pregnancy, women commonly experience an increase in appetite, with nearly one‐third of the women mentioning they consumed more and smaller portions throughout the day. Pregnant women faced challenges in improving their diet quality due to physical well‐being issues. Common challenges included nausea and heartburn, leading women to restrict their food intake making it difficult to obtain adequate nutrients. In addition, women commonly face challenges, such as cravings and aversions impacted by hormonal changes. Some women developed aversions to healthy foods such as fish or vegetables. In addition, women frequently developed cravings for unhealthy sweet treats, although most women tried to resist indulging in these cravings, striving to find a balance in their diet. Finally, many women experienced temptations during pregnancy, such as the urge to snack or to resist undesirable food groups. These challenges highlight the complex relationship between physical well‐being and diet quality during pregnancy.

Pregnant women expressed various reasons for changing their dietary pattern during pregnancy. First, a key driver for many women was receiving trustworthy information or advice, particularly from sources such as the dietitian and the app *Zwangerhap*. The internet in general was identified as a source of conflicting information about nutrition during pregnancy. As a result, women valued the opportunity to receive personal guidance from dietitians. Second, pregnancy itself was a strong motivator to start eating healthier. Many women reported maintaining a relatively healthy diet before pregnancy but were motivated to optimize their diet quality during pregnancy to ensure adequate nutrition for their baby's health. Third, women's concerns about their physical appearance played a role in motivating the improvement of their diet quality during pregnancy. Some were driven by a desire to avoid excessive weight gain, particularly those who had previous pregnancies or were already concerned about weight gain before conception. On the contrary, others wanted to relieve the pressure of strict dietary control during pregnancy, arguing that weight gain is an unavoidable aspect of pregnancy or that obsession over a balanced diet can become overwhelming. For example, one woman stated:

PW9: […]on top of that, when you're pregnant you're very focused on the things you're not allowed to eat, you already get enough stress from that. And if at some point you have to start stressing about the things you do need to eat, eating just isn't fun anymore. So at some point, I let it go a bit.

Improving diet quality was accompanied by several challenges and strategies, despite the awareness of the significance of good diet quality and the motivation women felt because of the P4HP consultations and the baby's health. First, making substantial changes in dietary choices was often experienced to be difficult because of certain habits or dislikes, whereas many women found it easy to make small changes in dietary intake. Consequently, for most women, it was valuable to receive guidance from a dietitian to obtain targeted advice on tackling these challenges. Second, for women on maternity leave, disrupted daily routines posed challenges. Some women became more prone to unhealthy snacking more to boredom, while others used the opportunity to focus on healthier eating habits and meal preparation. Third, women perceived the family environment as both a challenge and a strategy for improving their diet quality during pregnancy. A prevalent coping strategy involved creating a healthy home food environment by avoiding stocking unhealthy foods. However, social events, such as holidays, birthday parties or dining out provided additional challenges. As women often take responsibility for the family diet, they consider the preferences of other family members. As a result, partners or children may tempt them to eat unhealthy foods for example because they prefer fast foods. In addition, if their partner or children dislike healthy foods, such as fish or vegetables, they avoid preparing them. The family environment also acted as a strategy, as some women started eating healthier to set a good example for their children or to provide them with adequate nutrition.

PW11: Yeah, what also really helped was that my boyfriend was really supportive. He is also into healthy things, so it's great that you don't have to do it all by yourself. He also says things like ‘let's make a salad’ or ‘let's just cook’ if I want something unhealthy for dinner.

PW16: I feel the responsibility to eat healthily, also for the other children. You set a good example. I really want them to enjoy everything, and also understand the value of healthy eating, not only now but also later.

Almost all women perceived their dietary intake as solely their responsibility, often taking charge of the grocery shopping, and cooking regularly. Women started to think about their diet quality and came up with strategies for independently improving it since they felt a strong feeling of responsibility for the health of their unborn child. Nine women reported dietary changes that were self‐initiated rather than prompted by a dietitian or midwife. These changes sometimes were based on their own perceived knowledge or from information they found online. For example, concerns about potentially inadequate intake of nutrients by their children led to these dietary adjustments. However, women did not want to be too restricted regarding their dietary intake, allowing some flexibility.

PW3: I think it is my responsibility. I'm pregnant, so I influence what he [the baby] gets in. So, I definitely think it's my responsibility.‬‬‬‬‬‬‬‬‬‬‬‬‬‬‬‬‬‬‬‬‬‬‬‬‬‬‬‬‬‬‬‬‬‬‬‬‬‬‬‬‬‬‬‬‬‬‬‬‬‬‬‬‬‬‬

While nearly all women viewed their dietary intake as their responsibility, three women explicitly mentioned their partner as sharing in this responsibility. Particularly shared meals and grocery shopping were perceived as joint responsibilities. Twelve women were supported by their partners regarding their dietary intake practically, such as by cooking or grocery shopping, and emotionally. Partners attended consultations, often reassured the women about their dietary intake, and encouraged healthy eating habits. However, one woman did not feel supported by her partner. During grocery shopping, she would sometimes become emotional when faced with foods she could no longer eat due to pregnancy food safety guidelines. In response, her (PW6) partner's dismissive remarks, such as ‘don't be so moronic’ made her feel judged and unsupported.

PW7: So yes, it is a shared responsibility, because we have dinner together. But the rest of the day, of course, it's my responsibility. So, it's a little bit of both.

Pregnant women who had not yet given birth expressed a desire to sustain a good diet quality postpartum and intended to follow the dietitian's recommendations recognizing the importance for their own and their children's health. All 14 postpartum women experienced responsibility for additional changes in dietary intake in the postpartum phase, for example when breastfeeding. As they recognized that having a baby is a life‐changing event, many women underestimated the impact of this period and reported the need to re‐establish a regular daily routine. Some reported that sustaining a good diet quality during the postpartum phase was more challenging than during pregnancy, due to factors such as increased appetite from breastfeeding, sleep deprivation, time constraints and irregular schedules. In addition, many women lacked external motivation to eat healthily for the baby, making them prone to reverting to previous eating habits. However, the prospect of future breastfeeding or providing nutritious meals for their child motivated some women to maintain a healthy diet.

PW11: After breastfeeding, I think [healthy eating] will continue because she will also start eating and you want her food to be healthy. And you also think about the better your immune system is, the fewer diseases you will pass on, so there's a motivation behind it.

### 3.2 Theme 2: Most Pregnant Women Evaluate the P4HP Programme Positively

Sixteen women experienced the P4HP programme as positive. Women expressed gratitude for the emphasis on healthy eating during pregnancy because they recognized their responsibility for the baby's health. The benefits of the P4HP programme extended beyond pregnancy; they contributed also to maintaining good dietary habits in the postpartum period. Almost half of the women perceived the consultations as a motivation to change their diet because they knew their diet would be a topic of discussion. This prompted them to reflect on and become aware of the significance of their diet quality.

PW4: Yes, and from then [the moment the midwife asked if she wanted to participate] I thought okay, then I'm just going to try to pay attention [on healthy eating] from now on. And the dietitian made it more clear what I'm doing it for.

The empowering style of consultation was experienced positively. Women received personalized nutritional advice tailored to their specific needs, which made them feel heard and understood. This strategy provided confidence and reassurance in their dietary choices, with the dietitian often confirming that they were generally eating well.

PW5: It was nice to hear from the dietitian that it's fine to snack in the evening […] and because I got some peace of mind from that, I have been snacking less.

Four women were neither distinctly positive nor negative of the P4HP programme, mainly due to their belief of having a diet of good quality requiring only minor adjustments. However, they acknowledged the importance of the P4HP programme for others who may benefit more from it. They made various suggestions for improvement, such as additional consultations with the dietitian, more in‐depth nutrition conversations with the midwife and group‐based implementation of the P4HP programme to foster mutual accountability and motivation.

Two women were not satisfied with the P4HP programme and consultation style, mainly due to differing expectations. One woman preferred more frequent consultations with the dietitian and a larger focus on knowledge transfer about maintaining a healthy diet during pregnancy. The other woman, who had maintained an extremely healthy diet before her pregnancy, was not satisfied with the P4HP programme since she preferred more extensive discussions and a stricter approach.

PW12: I thought if it [the P4HP programme] keeps me eating healthily during the whole pregnancy and I will be able to resist cravings, that would be great. But that wasn't the case. Maybe there needed to be more conversations […] and a better overall view, and for someone to explicitly say: ‘You absolutely need to resist that because it's not good for you’.

In total, 19 women recommended the P4HP programme to other pregnant women, regardless of their own experiences, whether positive, negative or ambivalent, with the programme. Although almost all women indicated that the P4HP programme was valuable to them, many women perceived that it could be more relevant to other pregnant women. For example, they suggested that the P4HP programme might be particularly beneficial for pregnant women with low SES, poor dietary intake or those unaware of their intake. Interestingly, most of these participants perceived themselves as already being aware of their dietary intake and their diet quality as healthy and varied. Therefore, some did not consider themselves as the target group for the P4HP programme. Nevertheless, they endorsed the P4HP programme, believing it could raise awareness among other pregnant women of their dietary intake.

PW13: The consultation with the dietitian was particularly memorable and helped me a lot. It was very relevant to me, which was funny because I also realized that maybe I wasn't really the target audience for the study. I know, or thought I knew, what good nutrition is and making conscious choices in that. I always have. I can imagine that the study is aimed more at people who have trouble making good choices and maybe need more support in that. Therefore, I thought I was not really the target group at all, but in the end, I got a lot out of it.

### 3.3 Theme 3: The Dietitian Plays a Key Role in Empowering Pregnant Women Towards Healthy Dietary Intakes

Participants experienced the role of the dietitian as very valuable and therefore perceived the consultations positively. The main reason for this positive perception was that the consultations heightened the awareness of all women, prompting reflection on their nutritional behaviour. Through these consultations, the dietitian provided new insights with specific and practical dietary advice, empowering participants to make healthier food choices. Additionally, half of the participants liked to be reassured by the dietitian about their nutritional behaviours and dietary intake, also because of the responsibility the women felt towards their baby's health.

PW19: The consultations made me extra conscious of what you're eating, when you're eating, and what you're doing it for. You already know that, and you do it, but you're extra mindful and extra careful.‬‬‬‬‬‬‬‬‬‬‬‬‬‬‬‬‬‬‬‬‬‬‬‬‬‬‬‬‬‬‬‬‬‬‬‬‬‬‬‬‬‬‬‬‬‬‬‬‬‬‬‬‬

Moreover, 15 participants experienced it as pleasant that the consultation helped them to gain more confidence in their dietary intake. This enhanced confidence was often due to the provided reassurance and confirmation by the dietitian. As a result, gaining confidence positively affected women's feeling of being in control of dietary intake.

PW12: I always had the fear of, what if I get fat and I can't get it off? Being too fat has always been a thing for me, so that's my insecurity. But the dietitian said, ‘You are already well on your way, just keep it up’. That was good to hear.

The empowerment aspect of the consultations was also experienced positively by the participants, as women experienced that their situation and capabilities were taken into account by the dietitian. In addition, some women appreciated being in control during the consultations. For example, they found it beneficial to address their challenges such as cravings and nausea, and discuss topics of personal importance during the consultations. In addition, the dietitian considered the individual nutritional needs of the women and provided, based on this, personalized information. This increased their nutritional knowledge, which was experienced positively. This personalized information mainly consisted of practical and concrete advice or strategies that were easily applicable. By giving such tailored guidance, the dietitian supported changes in diet quality for almost all women increasing their perceived control over their dietary intake. Women perceived the nutritional expertise of the dietitian as positive and trustworthy, appreciating the increase in their nutritional knowledge. Some considered the dietitian more trustworthy regarding nutritional knowledge than the midwife, as the midwife's knowledge was often perceived to be more basic, while the dietitian had received specialized education in nutrition.

PW11: She said, as your pregnancy progresses, more nutrients will go to the baby, leading you to build up a deficit. So it was nice to get some explanation and advice at the same time, because she gave me very concrete advice (…). That's what I like most, that you don't just get some advice or vague tips or things like that, but that it is very concrete.

Eight participants, especially postpartum women, mentioned a wish to have more consultations with the dietitian. These additional consultations could be valuable for evaluating the woman's dietary intake and reflecting on the advice given by the dietitian. Additionally, considering that the nutritional needs of pregnant women vary across pregnancy trimesters and dietary preferences may change throughout the pregnancy, some women advocated for a consultation with the dietitian per trimester. These extra consultations would allow the woman to ask the dietitian more questions, discuss challenges throughout pregnancy, serve as a motivation for maintaining dietary changes and provide more reassurance. An additional consultation dedicated to, or during, the breastfeeding phase with the dietitian would be appreciated since nutritional needs change again during this phase. This was neither discussed during the dietitian nor the midwife consultations. Specific barriers during the postpartum phase were experienced, such as lack of time, lack of sleep and an increased appetite. Therefore, it would be appreciated to be able to discuss such challenges and barriers during both pregnancy and postpartum periods with the dietitian.

PW18: (…) Especially when your stomach is getting smaller, you just can't eat as much, but it's still important to get good nutrients in, and I think that the transition between your second and third trimester might be a good time to have another conversation with a dietitian to talk about, ‘I'm going to eat less now, how can you best accommodate that, what kind of product can you eat best?’ Especially in your eating pattern at that time.

PW9: For me eating after pregnancy was maybe even more challenging, because when you're breastfeeding you need a lot of extra calories. I noticed that in the first 4 weeks after giving birth, I started eating unhealthier at that point, purely to get your calories in.

Six women in this study had two consultations, instead of one, with the dietitian. Four of them explicitly reported this extra visit as helpful. Having two consultations with the dietitian was experienced positively as the second visit allowed for reflection on the first consultation. In addition, the follow‐up consultation allowed them to tackle any challenges encountered since the first consultation, and explore additional strategies to improve diet quality. One woman preferred scheduling the second session later in pregnancy because of evolving nutritional challenges.

PW17: Having two dietitian consultations was good, because in the first one, we talked more about what I could do better, and in the second one we talked about whether it worked. And if it didn't work out, you can get new tips on how to do it. Though another consultation might be helpful, because you can fall back into your old habits.

### 3.4 Theme 4: Midwives Support Pregnant Women in Maintaining Dietary Improvements

All participants experienced the consultations with the midwife positively, as they often reminded them to stay aware of their dietary intake and any adjustments made. This motivated women to maintain a healthy dietary intake. Women appreciated being reassured or confirmed by the midwife, and some women explicitly stated that these interviews made them more aware of their dietary choices. They reported experiencing control over the discussion topics during the consultations. Participants also perceived that the midwife considered their personal situation, challenges and abilities when discussing dietary intake.

PW5: During the conversations with the midwife, it came up of course and it was a moment of ‘oh yeah, that's what the dietitian said, I'll pick that up again.’ I experienced that as positive.

PW11: You had to think for yourself beforehand about your questions, so that helps you think about what my questions are, or what is bothering me. And I thought that that was the case for both the midwife and the dietitian, that they did ask ‘What are your questions or what are you still struggling with, what do you find difficult or what is going well?’ So that was good.

While the participants generally experienced consultations with their midwife positively, many perceived them to have a smaller impact on their dietary intake than the consultations with the dietitian. During reflection consultations around the 22nd and 32nd weeks of pregnancy, dietary intake was often briefly addressed, before the midwife continued with the remainder of the consultation and physical check‐ups. In general, the consultation with the midwife was remembered in less detail compared to those with the dietitian. However, the midwifery consultations were experienced as helpful reminders of topics discussed with the dietitian, leading to increased awareness and sustained improvements in diet quality:

PW15: The two follow‐up consultations with the midwife were very short conversations. She asked a few times how it had been with the dietitian and if I had gotten anything out of it. (…) But they didn't amount to much. It was more like, ‘did it help you’, ‘yes’, ‘how exactly’, and she wrote that down and that was it.

Three participants experienced insufficient coordination between the midwife and the dietitian. For example, similar questions were asked during the consultations with both healthcare professionals. These women expressed that the consultations with the midwife could have been more valuable had she been better briefed on the content of previous dietitian consultations. This would have allowed more meaningful reflection and discussion regarding participants’ dietary intake and changes.

PW14: It didn't seem like the midwife was aware of the information from the dietitian, I had to tell them again what I had discussed with the dietitian because she asked about that, but then I had to explain it again. If the midwife had been more aware of what was discussed, I think they could go deeper into other things, if they had thought about that beforehand.

## 4 Discussion

This study explored pregnant women's experiences with the P4HP programme and its impact on dietary behaviours during pregnancy. All participating women made dietary improvements during pregnancy, primarily promoted by their baby's health. The P4HP programme was positively evaluated by most participants, with women particularly valuing the personalized nutritional guidance from dietitians and the supportive role of midwives in maintaining dietary changes. Importantly, the programme successfully employed an empowerment approach, increasing women's awareness and confidence in making dietary choices through reassurance and confirmation from midwife and dietitian.

Pregnant Dutch women participating in the P4HP programme significantly improved their diet quality [31]. Women in this study reported increased consumption of dairy products, fruits, vegetables, fish and water, while omitting unsafe foods and making changes to sugary food intake. However, women faced challenges from conflicting online information. This was also shown previously in a study by Super et al. [25] that highlights the significant role of dietitians in raising awareness about the importance of diet and promoting meaningful dietary changes. A systematic review by Hanifi et al. [45] showed a lower prevalence of low birth weight and preterm infants when dietitians were involved in antenatal care. Given the positive impact dietitians can have on dietary change during pregnancy [46, 47], and considering these reported challenges, additional dietitian consultations during pregnancy and in the postpartum phase could be beneficial to address nutrition challenges during these critical periods.

Both dietitians and midwives contributed significantly to women's experiences of empowerment. Dietitians provided detailed nutritional guidance, while midwives reinforced and supported preferred dietary changes over time. The strong positive response to dietitians’ personalized guidance likely reflects their professional training in MI [25, 28, 29]. This complementarity suggests that the collaboration between differently trained professionals may strengthen the programme's effectiveness. Although midwives have rarely been involved in behaviour change programmes [33], our findings indicate that their contributions to the P4HP consultations were valuable. Midwives helped increase pregnant women's awareness and strengthened their dietary confidence through reassurance and confirmation. As Szwajcer et al. [6] argue, ‘hot awareness is more than cold knowledge’—when women value healthy eating and receive thoughtful guidance, this ‘hot’ awareness leads to more active monitoring of eating habits.

However, midwives face several barriers in providing nutritional support. McCann et al. [23] found that midwives often lack confidence in delivering pregnancy‐specific dietary advice. There is limited information on nutrition training in midwifery education programmes [48]. Consequently, pregnant women in developed countries often receive inadequate nutrition advice [49]. Despite these challenges, our findings suggest that when midwives collaborate with dietitians, their ongoing supportive relationship with women can effectively reinforce and sustain the specialized dietary guidance provided by dietitians. Our findings align with Abayomi et al. [50] in demonstrating that women want positive, practical dietary guidance focused on achievable actions rather than dietary restrictions. This emphasizes the need for collaborative care between dietitians and midwives, where dietitians provide their specialized nutritional expertise while midwives leverage their unique patient relationship to reinforce and sustain dietary changes. Together, this combined approach creates an empowering environment that supports pregnant women in achieving and maintaining positive dietary changes.

Participants reported heightened awareness and confidence about their dietary intake because of the P4HP programme, indicating they felt empowered to adopt a healthier diet. This increase in confidence often came from reassurance and confirmation by health professionals. These experiences align with Tengland's [51, p. 90] definition of empowerment in a professional setting: ‘A change (internal or external to the person) is an increase in empowerment if (if and only if) it is an increase in the person's control over the determinants of her quality of life, through (necessarily) an increase in either health (e.g., through self‐confidence, self‐esteem, self‐efficacy, autonomy), or knowledge (self‐knowledge, consciousness‐raising, skills development, competence), or freedom (negative or positive).’ While most women appreciated this empowerment‐based approach, a minority preferred a more strict and educational approach, with dietitians providing detailed information or advocating for specific dietary restrictions. This variation in preferences aligns with Tengland's [51] statement that empowerment can be achieved through various processes, such as recommending, suggesting, encouraging and informing, depending on the caregiver's influence and the power balance between caregiver and client. Therefore, we recommend that both dietitians and midwives tailor their empowerment strategies to individual preferences, flexibly adapting their approach both within and between consultations to enhance the effectiveness of nutrition guidance during pregnancy.

This research advances both scientific understanding and clinical practice by demonstrating the effectiveness of an empowerment approach to improving diet quality during pregnancy. While education has traditionally dominated health behaviour interventions for pregnant women, our findings support previous research indicating that knowledge alone is insufficient [33, 52, 53]. Currently, midwives’ nutrition guidance typically focuses narrowly on food safety issues rather than comprehensive healthy eating strategies [22], despite evidence that consistent attention to healthy eating can foster sustained nutritional awareness and eventually lead to automatic responses [54]. While midwives are ideally positioned to provide ongoing nutritional guidance, they often lack the necessary resources, knowledge and time [48]. These limitations underscore the need for innovative approaches to nutrition communication in maternal healthcare.

A promising approach to enhance nutrition support in antenatal care is CenteringPregnancy, which offers empowering group sessions that provide peer support and motivation for maintaining healthy dietary habits [55, 56]. However, recognizing that group consultations may not suit all women's comfort levels or communication preferences, we advocate for a flexible, collaborative model between midwives and dietitians that can accommodate individual needs. This collaboration allows midwives and dietitians to leverage complementary expertise [20, 25], particularly when supported by physical proximity to facilitate access, and reduce barriers to nutritional guidance.

### 4.1 Study Strengths and Limitations

The first strength of this research is the qualitative design used to study pregnant women's experiences with and perceptions of the P4HP programme, providing a detailed understanding of the mechanisms behind changes in diet. Second, achieving data saturation enhances the robustness of this research. Third, conducting all interviews in the women's homes provided a confidential and familiar environment allowing women to feel secure and share personal information, thereby facilitating a deeper understanding of experiences and perceptions [57]. Fourth, the information discussed during the P4HP consultations corresponded to information discussed during the interviews for all but one woman. This suggests that the information shared during the interviews was reliable and that the women were open and honest during the interviews.

A limitation of this study is the restricted generalizability of its findings due to the characteristics of the participant group. The study primarily comprises a group of relatively highly educated and motivated individuals. Many participants reported maintaining a fairly healthy and varied diet before pregnancy and perceived themselves as already being well‐informed about their dietary intake. As pointed out by several interviewees, the fact that pregnant women with low SES experience more barriers and have more to gain as they tend to have lower diet quality to begin with, this group may particularly benefit from the P4HP programme. In line with this, a limitation of our study was the requirement for participants to follow a traditional Dutch dietary pattern, which may have excluded individuals with migrant backgrounds who maintain different cultural food practices. This criterion possibly limited the diversity of experiences and perspectives captured in our interviews. As a result, the findings derived from this specific sample may not be broadly applicable or reflective of the entire study population, thus limiting the external validity of the study. Future research could explore the experiences of pregnant women from various cultural and economic backgrounds with the P4HP programme, as their needs, challenges and preferences may differ from our study population.

Before conducting the interviews, it was expected that employing a visual aid and allowing the interviewee to contribute to a timeline would foster a collective process triggering collective memory and enhancing the participant's ownership [37]. However, a limitation was that the timelining tool did not always have the intended effect because women's experiences and changes in diet quality were already well‐established in their minds due to the relatively short and unique event of pregnancy. In addition, the researchers were more active in completing the tool than the participants, who frequently forgot to record important topics. With time‐lining often being used for life history research [37], the relatively short duration of pregnancy may have contributed to this ineffectiveness. Another reason was that some participants were occupied with caring for their babies, making it impractical for them to write on the timeline. Nevertheless, the tool was helpful for the researchers to ask questions about the different consultations in chronological order, thereby structuring the interview, and enabling more in‐depth explorations of consultations separately. In future research, timelining could therefore be a valuable aid to assist interviewers.

### 4.2 Implications for Practice and Future Research

Based on our findings, we recommend several key changes to improve antenatal care. First, we advocate for the integration of dietitian consultations into standard antenatal care, ideally each trimester of pregnancy and extending into the postpartum period. The complementary roles of dietitians and midwives highlighted in our study could inform updated antenatal care guidelines, emphasizing the importance of structured nutrition consultations throughout pregnancy. Implementing these recommendations will require policy changes, particularly regarding insurance coverage for nutritional care, and should be supported by further research. Future studies should evaluate the cost‐effectiveness of integrated dietitian services, explore strategies for establishing sustainable collaborative care models and assess the long‐term impact of the P4HP programme on maternal and child health outcomes. This comprehensive approach has the potential to significantly improve nutritional support for pregnant women and ultimately enhance maternal and child health outcomes.

## 5 Conclusion

This study investigated the experiences of pregnant women who participated in the P4HP programme, including reported changes in diet quality, factors influencing these changes and the pregnant women's perceptions of the P4HP programme. Overall, pregnant women perceived the P4HP programme positively as they raised awareness, reassurance and confirmation regarding the quality of their dietary intake, thereby increasing their confidence. Even though all pregnant women reported making changes to their diet during pregnancy to improve their and their baby's health, they encountered challenges in sustaining these changes, including after childbirth. Although the consultations with the midwife motivated them to sustain changes, it was the dietitian who provided personalized nutritional advice that made women aware of their diet quality, offered them new insights, and boosted their confidence and reassurance. The P4HP programme demonstrates the potential of collaborative, empowerment‐based approaches to improve prenatal nutrition. While our findings advocate for the integration of dietitian services into routine antenatal care, successful implementation will require addressing systemic barriers and ensuring accessibility for all socioeconomic groups. Based on the findings, we recommend incorporating dietitian consultations as part of standard maternal healthcare to promote lasting improvements in diet quality.

## Author Contributions

All authors conceptualized and designed the study. S.P. and F.L. carried out the interviews and carried out the analyses. R.M.L., S.P., F.L. and A.W. repeatedly discussed the coding process. R.M.L. wrote the manuscript. All authors contributed to review and editing before submission and approved the final manuscript.

## Ethics Statement

The research was approved by the Medical Research Ethics Committee Utrecht on September 21, 2021. Participation in this study was voluntary and participants were free to withdraw at any time without consequences or the need to disclose their reasons. Participants provided written informed consent before each interview.

## Conflicts of Interest

The authors declare no conflicts of interest.

### Peer Review

The peer review history for this article is available at https://www.webofscience.com/api/gateway/wos/peer-review/10.1111/jhn.70027.

## Transparency Declaration

The lead author declares that this manuscript is an honest, accurate, and transparent account of the study being reported. No important aspects of the study have been omitted.

## Supporting information

Supporting information.

Supporting information.

## Data Availability

The data that support the findings of this study are available on request from the corresponding author. The data are not publicly available due to privacy or ethical restrictions.
