# Supplementary material for: Pregnant Women's Experiences With a Collaborative Midwife‐Dietitian Empowerment Programme to Improve Diet Quality
Source: J Hum Nutr Diet. 2025 Feb 11;38(1):e70027. doi: 10.1111/jhn.70027 (PMC11813828; doi:10.1111/jhn.70027)
Supplement: Supplementary file 1 — Supporting information. [file JHN-38-0-s002.pdf]

Supplementary file 1: Handbook Power 4 a Healthy Pregnancy programme

Implementation in the midwifery practice

Manual for midwives and dieticians

POWER4 A HEALTHY PREGNANCY

Introduction

This document serves as a manual for implementing the Power4HealthyPregnant program in the midwifery practice. This manual is intended for midwives and dietitians. There are two types of practices in this study: intervention practices and control practices. In this document you will find the most important information for **intervention practices** to implement the program. There is a separate document for control practices.

The **structure** of this manual is as follows: In Chapter 1 you will find information about the background it of the Power 4 a Healthy Pregnancy program. Chapter 2 outlines the program. Chapter 3 deals with the recruitment of pregnant women and Chapter 4 with the implementation of the program. Chapter 5 describes what participation means for midwives and dietitians in terms of time investment, collaboration and administration. Chapter 6 describes the questionnaires completed by participating women. The final chapter 7 elaborates on a case study on the use of the conversation card. The appendix contains the conversation card.

Do you have a question to which you cannot find the answer in this document? Do not hesitate to contact the researchers, we will be happy to help you.

Susanne Cremers and Renske van Lonkhuijzen

[contact details]

WAGENINGEN UNIVERSITY & RESEARCH

Content

Supplementary file 1: Handbook Power 4 a Healthy Pregnancy programme 1
1. Why Power 4 a Healthy Pregnancy? 2
2. What does the Power 4 a Healthy Pregnancy program look like? 3
3. Roadmap for recruitment of participants 5
4. Roadmap of the Power 4 a Healthy Pregnancy consultations 7
   1st moment: Early information by the midwife 8
   2nd moment: Consultation with the dietician (~12 weeks of pregnancy) 13
   3rd moment: Reflection with the midwife (~22 weeks of pregnancy) 14
   4th moment: Reflection with the midwife (~32 weeks of pregnancy) 14
5. What does participation in Power 4 a Healthy Pregnancy mean for midwives and dietitians? 15
6. Description of conversation card 16
7. Example case study 17
References 22
Appendix 1: Conversation card 23

1. Why Power 4 a Healthy Pregnancy?

Nutrition affects maternal and child health across the life course[1]. Research shows that healthy nutrition in the first thousand days from conception (until the child's second birthday) is also important for the child's health in (late) adulthood[2]. Pregnancy is a promising time for lifestyle changes because women see the importance of healthy eating for their own health and that of their child[3]. On the other hand, especially during pregnancy, women experience challenges around nutrition, such as food aversions and nausea[4].

Power 4 a Healthy Pregnancy is a program in which midwives and dietitians and pregnant women work together on healthy eating. Nutrition is discussed in the program at four points during pregnancy.The goal of Power 4 a Healthy Pregnancy is for the pregnant women to improve their nutritional quality during pregnancy through empowerment.

Empowerment is the ability of individuals or groups to enhance capabilities, critically analyze situations and take actions to improve these situations[5]

The relationship of trust between the midwife and the pregnant woman plays an important role here. The program was developed in co-creation with pregnant women, midwives and dietitians and other stakeholders[6,7].

2. What does the Power 4 a Healthy Pregnancy program look like?

What does the Power 4 a Healthy Pregnancy program look like? The study has an intervention group and a control group, as shown schematically in Figure 1. This paper is about the relevant papers for the intervention group - the group working with the Power 4 a Healthy Pregnancy program. Power 4 a Healthy Pregnancy can be implemented in an individual course or integrated into Centering Pregnancy (CP). This chapter gives a brief overview of the research design and materials you can use. Chapter 4 provides a more detailed description of the interviews.

Baseline measurements (T0)

When a woman decides to participate in Power 4 a Healthy Pregnancy and has signed the informed consent form, she completes a number of questionnaires on nutritional quality and psychosocial health prior to the program. An explanation of these questionnaires can be found in Chapter 6.

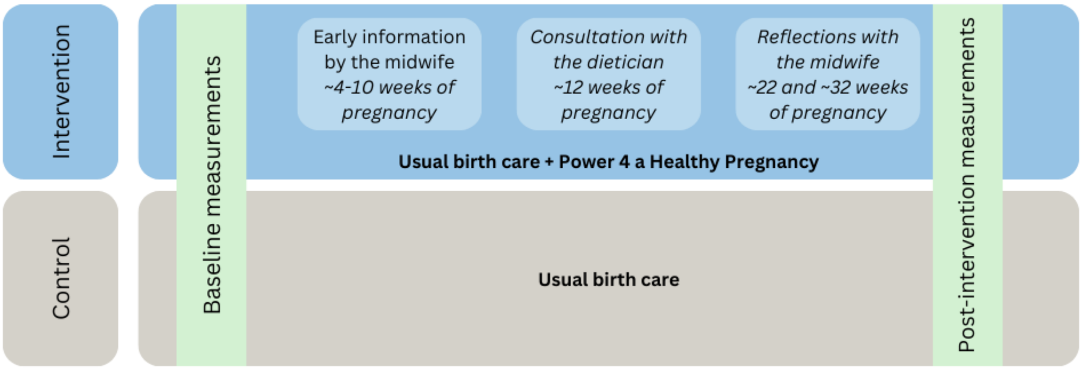

Figure 1: Study design of Power 4 a Healthy Pregnancy

During the program, the pregnant women have a conversation about nutrition with the midwife or the dietician on four occasions. The first meeting with the midwife takes place in the first trimester of pregnancy. Exactly when that is depends on how your practice works. The conversation lasts 10-15 minutes.

The second conversation about nutrition takes place with the dietician, around the 12th week of pregnancy. As a nutrition expert, the dietitian can go deeper into what the pregnant person is struggling with, such as nausea or fatigue. This conversation takes 30-45 minutes.

Around 22 weeks and 32 weeks, the pregnant woman reflects with the midwife on her nutritional intake of the past weeks. The midwife supports and encourages the pregnant woman to come up with her own solutions and possibilities that suit her and her abilities. This increases a woman's own effectiveness. These reflection moments last about 10 minutes.

Post-intervention measurements (T1)

At the end of the program, women again complete the questionnaires on nutrition and psychosocial health. We compare the outcomes with those of the control group. This allows us to see if anything has changed because of the program.

Materials

In the Power 4 a Healthy Pregnancy program, you can use the following materials. These materials will be covered and explained throughout this manual.

For the midwife/dietitian:

- Manual for midwives and dietitians (this document).

- Flow chart for recruitment and implementation of the P4HP programme (Figures 2 and 3)

- The interview card (found in the General Documents folder in your practice's Microsoft Teams environment and also in Appendix 1 of this document)

- The registration and peer communication document (found in the participant folders in the Teams environment of your midwifery practice)

- Sample case study for the first interview (Chapter 7)

For the pregnant woman:

The conversation card (Appendix 1)

- After the consultation you can give the completed discussion card. On it are noted goals and on the back tips on where the pregnant person can find more information.
- The back of the conversation card refers to the following information:ZwangerHap
    - The Nutrition Center's app on eating safely during pregnancy www.voedingscentrum.nl/zwanger
    - Women can find information on healthy eating during pregnancy here www.youtube.com/c/voedingscentrum
      Prefer to watch a video? Here you will find information on healthy and safe eating during pregnancy, breastfeeding and also recipes.

3. Roadmap for recruitment of participants

Recruitment of subjects for Power 4 a Healthy Pregnancy is done by midwifery practices. From our conversations with practices, it appears that each practice operates differently. Discuss with your colleagues which method best suits your practice. The flow chart below (Figure 2) can help you find the right process for recruitment. Have questions? Let us know, we'd love to think along with you.

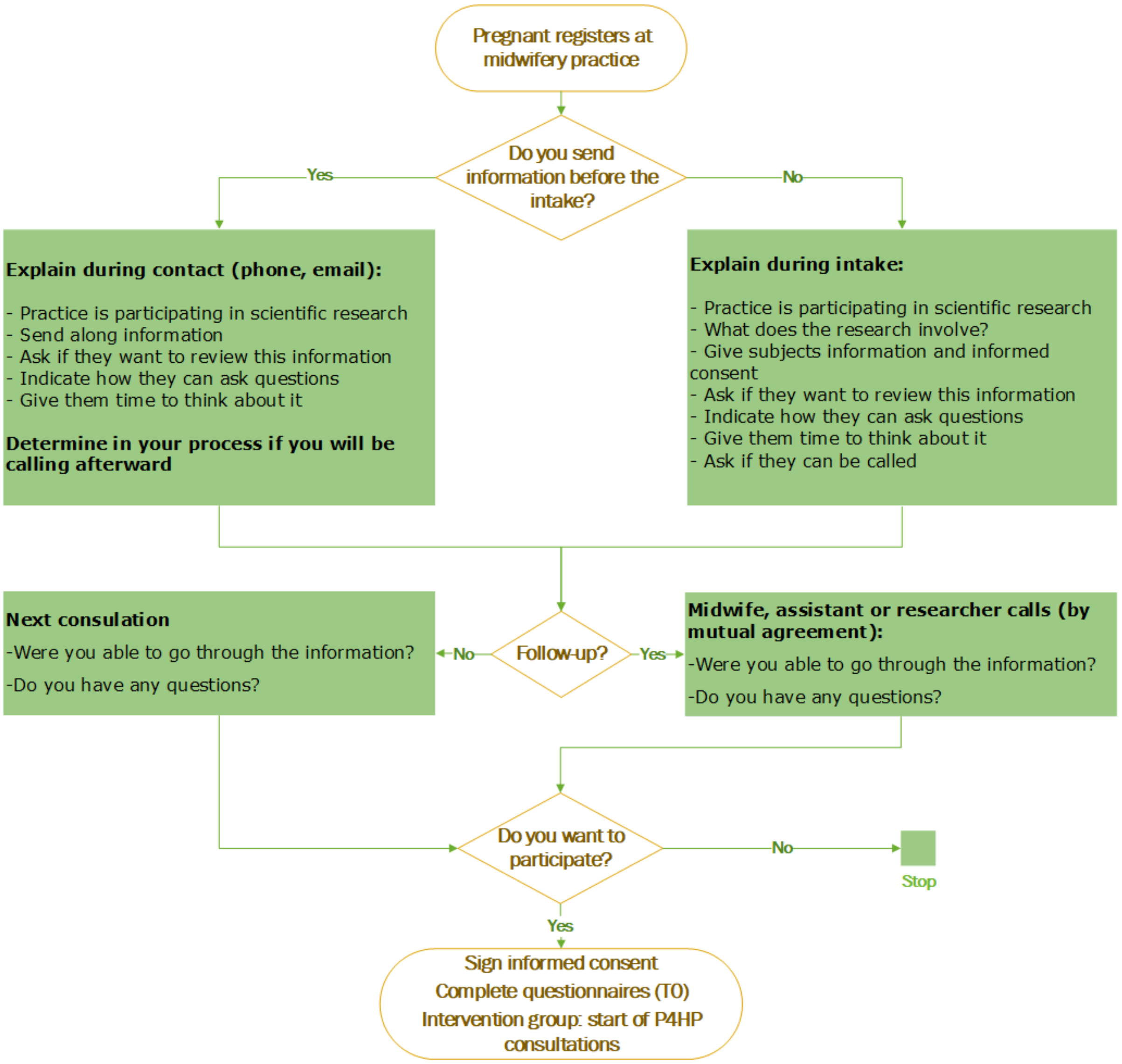

Figure 2: Roadmap for recruitment of participants

What is important in the recruitment process?
- The woman should have the opportunity to ask questions about the study
- The woman must have time for reflection to decide whether or not to participate in the study
- The consent form and questionnaires must be able to be completed before the first interview about nutrition

What is important during recruitment?

- Explain that the practice is participating in scientific research and that this is the intervention group.

- Explain what participation means for the woman.
- Provide the trial information and consent form or send it digitally.
- Come back to the study at the next contact. Ask if the woman has thought about whether she wants to participate and if she has any questions. Discuss with your colleagues whether to do this face to face, by phone or by mail.
- Does the woman decide to participate? Then have her sign the consent form and send it to the researchers. The woman will also receive a copy of this.
- Send the invitation to the woman to complete the questionnaires.

For your information:
- The questionnaire is sent from "de Eetscore". The questionnaire is specifically developed for during pregnancy. Professional guidelines, scientific articles and reports form the basis of the Eetcore questionnaire and advice. More information can be found here: http://www.eetscore.nl/
- De Eetscore is also the sender of the e-mail the women receive. You can prepare the participating woman for this.
- The mail the participating women receive contains both and invitation to the first part of the questionnaire (de Eetscore), and the second (questionnaire on empowerment, health and personal data). A link to an online questionnaire will take the women to the second part. Please communicate to participating women that they should complete both questionnaires.

4. Roadmap of the Power 4 a Healthy Pregnancy consultations

Has the woman signed the signed consent form (informed consent), has this form been uploaded into Teams and have the questionnaires been completed? Then you can start implementing Power 4 a Healthy Pregnancy. Below you will find the steps of the program incorporated into a flowchart (Figure 3).

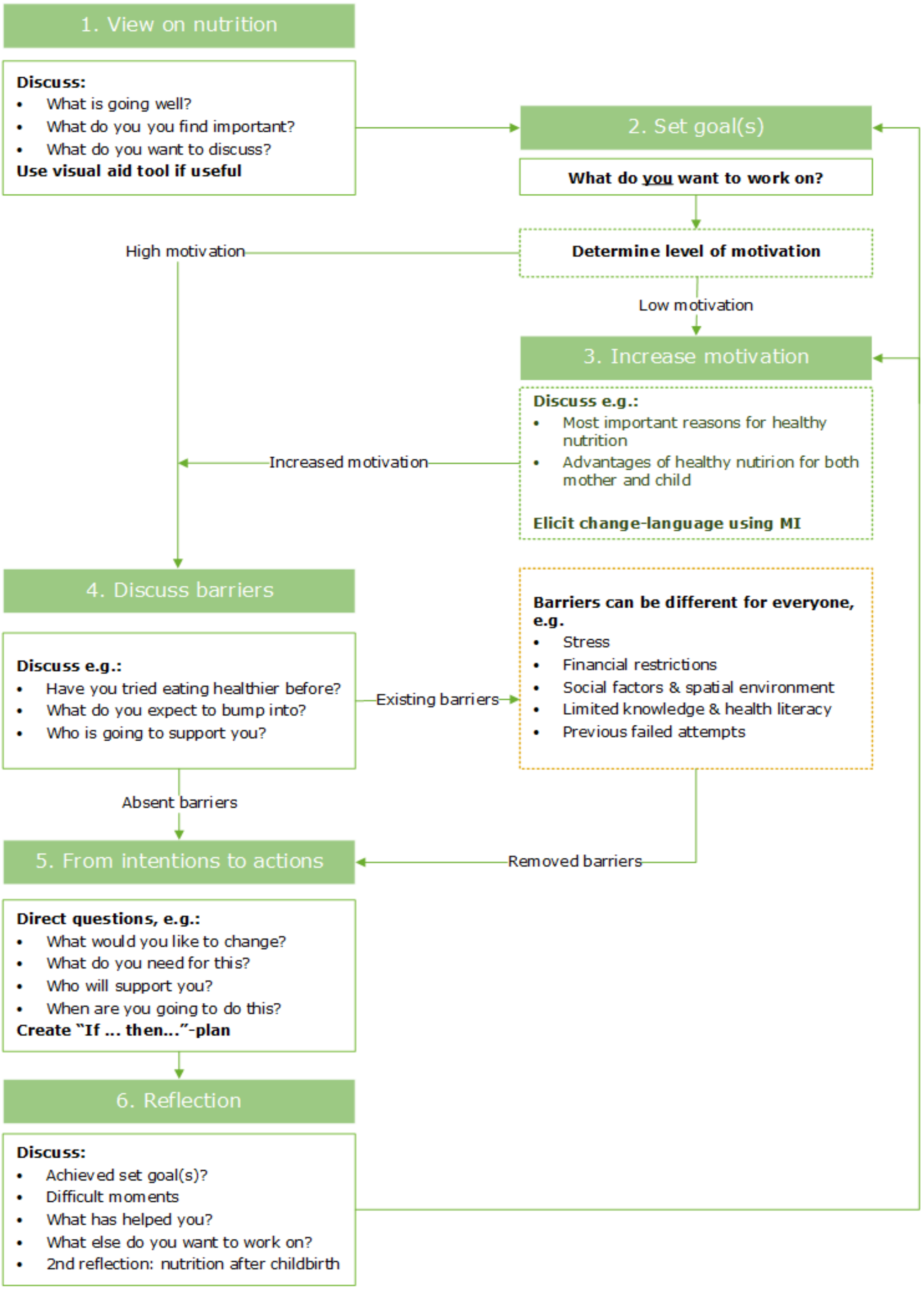

Figure 3: Flowchart Power 4 a Healthy Pregnancy consultations

You can go through the first 5 stages in the early education by the midwife (1st moment) and the consultation with the dietitian (2nd moment). The contact moments are detailed below.

1st moment: Early information by the midwife

What tools do you have for this conversation?

- *Visual conversation card (Appendix 1)*
- *Flow chart (Figure 3)*
- *Registration and peer communication document*

1. View on nutrition

Summary

Content: Provides information about the woman's motivation

Purpose: To estimate the woman's motivation

When: Early information

Discuss what the pregnant person herself thinks is going well regarding nutrition, what is important to her and what she would like to discuss. In the research setting, you can ask the woman why she would like to participate in the study, or refer to the questionnaire about nutrition she filled out. Again, you can get information about her motivation from this. Sample questions are:

"Can you tell me why you would like to participate in the study?"

"You have already thought about your nutrition in the past time when filling out the questionnaire. Would you tell me what you personally think is going well in your nutrition?

"I am very curious about what you think is important about eating during pregnancy. Could you tell me something about this?"

Important side note for the first consultation:

We understand that time for this first consultation is scarce. With some women, the steps are quicker to complete than others. We rely on your skills to sense what needs to be talked about and do what is possible within the time.

By doing this, you encourage the woman to look at her nutrition in a positive way. Give compliments and affirm her in her abilities to provide a healthy diet.

Then you can use the conversation card (Appendix 1) to let the woman decide what to continue the conversation about. You could use it in the following way:

"During pregnancy, a varied and healthy diet is important and a number of topics require special attention. On this card you will see different topics. Would you like to take a look at these and check off what you would like to talk about? You may check off multiple topics."

Checking topics works to increase commitment. Many pregnant women will check "What better not to eat". It makes sense that there will be questions about this. Motivate them to check multiple topics. This helps you find out what the woman is interested in and allows you to gauge her motivation. Ask out why she checked certain topics. By asking what she wants to talk about first, you leave control of the conversation with the woman:

"I see you checked 'vegetables,' 'snacks/ready-to-eat,' and 'what's better not to eat.' What makes you choose these topics?"

"What do you want to talk about first?"

From here you can see where she is open to change.

2. Set goal(s)

Summary

Content: Discuss what the woman wants and can change

Purpose: Agree on a concrete and achievable goal

When: Early information and consultation with dietitian

You can briefly summarize what the woman told you in step 1. Make the connection between what she thinks is important and what she wanted to talk about. You could do this in the following way:
"You indicate that healthy eating is important to you because you want to take good care of the baby. Good that you realize that healthy food is good for your child's development and also contributes to an enjoyable pregnancy. We have now talked about snacks and ready-to-eat, about vegetables and what you really should not eat during pregnancy. The latter goes very well with you. You say that you eat too few vegetables and too much ready-made food. What would you most like to change in the coming weeks?"

Let the woman herself write down on the conversation card what is going well and what she would like to change, this increases commitment. Do you feel that noting yourself is not successful? You can also ask if the woman wants to write it down herself or if she would rather you do it for her.

3. Increase motivation

Summary

Content: Indien nodig verhogen van motivatie

Purpose: De vrouw helpen haar redenen om gezonder te eten helder te krijgen

When: Early information and consultation with dietitian

In this manual in the example sentences "eat healthier" is used as the goal. In practice, you can use instead instead what the woman wants to change.

Make an assessment based on the conversation so far of how motivated the woman is. If the woman is highly motivated you can skip step 3 (increase motivation). Then confirm the woman in her motivation and proceed to step 4 (discuss barriers). Is motivation low try to increase it.

You can use the scale question about motivation:
"If I ask you how motivated you are to eat healthier, where do you rank on a scale of 0-10?"

Then you can ask the follow-up question:
"What makes it a 4 and not a 0?"
"What do you need to make a 4 a 6?"

Chances are slim that the women participating in the study are not at all motivated to eat healthier. Still, a woman may not yet be completely clear on why she should eat healthier. Discuss motivational information to stimulate the thought process. You can ask what the woman already knows about healthy eating during pregnancy and then ask for permission to provide additional information.
"May I explain a little more about this?"
"Do you mind if I tell you a little more about it?"

In education, connect to what the woman has previously indicated is important. You can emphasize direct benefits of healthy eating for mother and child[8]:
- Beneficial to the baby's physical and cognitive development
- Less chance of preterm birth
- Beneficial for preventing complications during pregnancy, such as gestational diabetes, pre-eclampsia or high blood pressure
- Healthy weight gain
- More energy, which is beneficial during childbirth

You might ask the following questions after this:
"Hearing this like this, what do you think?"
"What would be the most important reason for you to eat healthier?"

Next, pay attention to recognizing change language. Did you succeed in increasing motivation? Then move on to step 4 (discuss barriers).

If this step is the maximum feasible for this conversation, you can move to the next step of the program, the consultation with the dietitian. Hand out the conversation card and point the woman to the tips for more information on the back.

4. Discuss barriers

Summary

Content: Discussing problems the woman anticipates if she wants to eat healthier and mobilizing support from her immediate environment

Purpose: Increasing women's confidence that they can overcome these problems

When: Early information and consultation with dietitian

All change is difficult. If the woman prepares herself for challenges she will encounter, it will be easier to sustain the change. Therefore, pay attention to barriers, for example, with the following questions:
"Does it seem difficult to you to start eating healthier?"
"Have you ever tried to eat healthier before?"
"Suppose you decided to start eating healthier, what do you expect to encounter?"

Change language

To increase motivation, you can apply techniques from **Motivational Interviewing**. Provoking change language is one of these techniques. Change language indicates that someone is making a move toward change. If you know how to recognize change language, you can add a twist to the conversation. Examples of change language include:

- **Disadvantages of current situation:**
"I've been craving chocolate so much lately, but I've already gained 5 pounds."
- **Benefits of change:**
"I have heard once that healthy eating is important for the baby."
- **Intention to change:**
"I should/would like to watch my diet more, but..."
- **Ambivalence:**
"On the one hand..., but on the other hand..."
- **Optimism about change:**
"Now that I am pregnant I do think I will manage to drink less energy drinks."

The scale question of importance can help elicit change language: *"Looking at eating more vegetables, how important is that to you on a scale of 0-10?"*

Then you can ask: "Why is it a 4 and not a 0?" or "What do you need to turn a 4 into a 6?"

You can use the scale question about confidence to increase self-efficacy:
"Suppose you decide to start eating healthier, how much confidence do you have on a scale of 0-10 that you will succeed?"
Then ask through to increase confidence and remove barriers:
"What makes it a 4 and not a 0?"
"What do you need to turn a 4 into a 6?"

Information obtained in the earlier course of the general intake interview may also be relevant in the context of nutrition. Possible barriers to healthy eating include:
- Physical and mental complaints
- A lot of stress
- Domestic violence
- Financial constraints

- Social and spatial environment
- Knowledge and health skills

Help the woman remove barriers and mobilize support from her immediate environment:
"How can you reward yourself?"
"What do you need to start eating healthier?"
"Who will support you to start eating healthier (and who won't)?"

Tip:

Midwives and dietitians can take the free

e-learning **Goedkoop Gezonde Voeding** [Cheap healthy eating] (10 credits). This way you can guide women with limited financial options to eat healthy even better.

5. From intentions to actions

Summary

Content: Formulate a concrete goal

Goal: Putting intentions into action

When: Early information and consultation with dietitian

Once motivation and barriers are above water, it is important to translate good intentions to eat healthier into action. Support the woman to make an achievable goal as concrete as possible within her capabilities by asking pointedly:
"What exactly are you going to do?"
"How will you organize it to start eating healthier?"
"When are you going to do this?"

An "If ... then ..." plan can help a good intention succeed. For example:
"If I get hungry while watching TV, then I'll have a piece of fruit."

Have the woman write down the goal and the "If.... then..." plan on the conversation card.

Sometimes the situation is so complex that it is difficult to work on healthy eating. In that case, leave it at that and make a note in the file. You can then return to it at a later time.

Based on this conversation, book a consultation with the dietitian. Make sure that the dietitian has the results of the questionnaire and a short report available to properly follow-up. At least note in your record what/or was discussed:
- What is going well
- What does the woman want to change
- Possible barriers
- What goal/"if.... then..." plan has the woman formulated
- Details of the conversation

2nd moment: Consultation with the dietician (~12 weeks of pregnancy)

What resources do you have for this conversation?

- The record of the first conversation with the midwife
- Registration and peer communication document
- Visual conversation card (Appendix 1)
- Flow chart (Figure 3)

In this conversation you will have 30-45 minutes to go deeper into goals, motivation and barriers. You will have the midwife's report from the first meeting and the results available to you. This eliminates the need for you to do an extensive nutritional history. This conversation begins with step 6, a brief reflection.

6. Reflection

Summary

Content: Guidance for women in reflection
Purpose: Increase own effectiveness. Women are more likely to achieve long-term goals if they realize the effects of their own actions on outcomes.
When: Consultation with dietician, reflection moment 1 (22 weeks) and reflection moment 2 (32 weeks)

Come back to the goal from early information with the midwife and inquire about progress. The goal is for the woman to gain insight into supporting and impeding factors and to find ways to deal with difficult situations. This will increase her own effectiveness.

Were you successful in achieving this goal?
Give compliments for this success and use this to review with the woman where there are further opportunities for a healthier diet. For this, you can follow the structure with steps 2 through 5 (set goal, increase motivation, discuss barriers, move from intentions to actions).

Did it not work out?
Ask what the woman ran into (barriers). Find out if the woman is motivated to work on the chosen goal. Is motivation still high? Then from step 4 (discuss barriers) you can see what is needed to still make the goal achievable. Is motivation low? See if you can increase motivation (step 3) or if you should start at step 2 (goal) to see what the woman does want to change. Go through the step-by-step plan to step 5, turning intentions into actions, and conclude with concrete agreements.

Changes may occur during the course of the pregnancy, for example, with regard to physical symptoms. Therefore, check to see if anything has changed since the last consultation that has created new challenges.

Link back to the midwife what was discussed. You can also use the intervention card for this. Note at least the following in your record what/or was discussed:
- Achieved/not achieved goals

- Challenges discussed
- New goals and agreements
- Possible barriers
- What goal/"if... Then..." plan did the woman formulate
- Details of the conversation

3rd moment: Reflection with the midwife (~22 weeks of pregnancy)

What tools do you have for this conversation?

- The records of the early education and consultation with the dietitian
- Registration and peer communication document
- Visual conversation card (Appendix 1)
- Flow chart

Revisit the conversation with the dietitian. Assist the woman through targeted questions in her reflection (step 6). Were the goals met? If yes, what and who helped achieve them? What more can and would the woman like to accomplish (steps 2 through 5)?

If no, what made it difficult? How can the woman handle certain situations differently in the future to still achieve the goals? Who and what does she need to do so (steps 3 through 5)?

Note in your record what/or discussed:
- Achieved/not achieved goals
- Discussed challenges and barriers
- New goals and agreements
- Possible barriers
- What goal/"if... Then..." plan did the woman formulate
- Details of the conversation

4th moment: Reflection with the midwife (~32 weeks of pregnancy)

The 4th conversation has the same reflective nature as the 3rd conversation. Revisit the previous reflective conversation. The woman is now already in the third trimester of her pregnancy so other situations may arise that interfere with healthy eating. Are there new things the woman is encountering? What options does the woman have in these situations?

In this conversation you can address nutrition after childbirth.

After the 4th conversation, the woman will receive another invitation to complete the questionnaires (T1). Remind her of this in the consultation.

Note in your record what/or discussed:
- Achieved/not achieved goals
- Discussed challenges and barriers
- New goals and agreements
- Possible barriers
- What goal/"if... Then..." plan did the woman formulate
- Details of the conversation

5. What does participation in Power 4 a Healthy Pregnancy mean for midwives and dietitians?

Participation in the study requires a time investment by the midwifery practice and the dietitian. In the intervention group, we assume about 1.5 hours per pregnant woman for the midwife and about 1 hour for the dietitian (vacation allowance). This time is for recruitment of participants, the interviews and administration (Table 1). What we expect from you for each time is detailed below.

Table 1: Expected time investment of the Power 4 a Healthy Pregnancy Program

Action	Time
Recruitment of participants	15 min
1st moment: consulation with midwife (~8-10 weeks)	15 min
2nd moment: consulation with dietitian (~12 weeks)	30-45 min
3rd moment: consulation with midwife (~22 weeks)	10 min
4th moment: consulation with midwife (~32 weeks)	10 min
Administration	20 min

Collaboration
In the Power 4 a Healthy Pregnancy program, collaboration of the different midwives in a practice and the dietitian plays an important role. It is important to discuss the implementation of the program and ensure proper registration and transfer. The intervention chart can serve as a communication tool between the different health care providers. You can find it digitally in your practice's Teams environment. Discuss what would be the best means of communication between the different midwives in your practice. Also discuss this with the dietitian you work with.

Administratie
We are working with Microsoft Teams in this study to exchange information and communicate with each other. This is secure and low-threshold. Your practice has a private channel in the Power 4 a Healthy Pregnancy team, to which only the researchers and your own practice have access. In this environment, each participant has her own folder for her documents.

If a woman decides to participate in the study have her sign the consent form:
- Scan the signed consent form and save it in the participant folder in the Teams environment.
- Once the consent form is uploaded, the researchers can send the questionnaires to the participant.
Inform us by a short post on the private channel that you have included a new participant. For example, "Participant 1401 included on 2-12-2021, first interview scheduled on 15-12-2021."

Important: Participants must complete the questionnaires prior to the first interview. Therefore, it is important that the researchers know when that interview is scheduled. This way, we can remind the participant to fill it out, should the need arise. After each conversation, complete the intervention card on the Teams environment. You will find this in each participant's folder. We may ask you to do a final interview with us to evaluate the Power 4 a Healthy Pregnancy program. This is completely voluntary.

6. Description of conversation card

In chapter 7 you will find an example case in which you can see how you can provide early information (1st moment) about nutrition and how you can use the conversation card (Appendix 1). On the conversation card you will find images of different food groups that are important during pregnancy. The choice of these food groups is based on the advice of the Health Council for nutrition during pregnancy[2]. Show the conversation card to the woman during the first conversation and have her tick several topics she would like to talk about. During the conversation you find out what the woman thinks is going well with regard to her diet, what she would like to change and what goal she wants to set for herself for the coming weeks. Checking topics by the woman and writing down goals increases the woman's commitment.

Motivational interviewing

You can use the techniques of motivational interviewing in your conversation. Motivational interviewing is generally well developed among midwives and dieticians. As a reminder, below you will find some general tips and sample questions for all conversations in this programme.

Tips

- Ask permission to talk about nutrition
- Question thoughts and ambivalence carefully
- Let your conversation partner do a lot of talking, especially at the beginning of the process
- Do not contradict your conversation partner, avoid discussions
- Ask why someone wants to change her behavior
- Ask what motivates someone to change their behavior
- Pay close attention to change language
- Only provide information that your conversation partner is ready for and that is relevant at the moment

Sample questions for increasing motivation

- What would you like to see different about your food?
- How important is that change to you?
- What makes you think this is important? • And what else?
- If you were to eat more/less..., what would it benefit you? What solutions have you already come up with for this? What else could you think of?
- Who benefits if you .... eat? (Win-win situation, not only the woman herself but also someone else benefits from it, in this case the baby)

If the woman herself cannot think of anything:

I do have some suggestions. Do you want to hear it?/Can I share it with you?

7. Example case study

Martine, 32 years old, married to Bart, and 7 weeks pregnant with her first child. Martine works full-time as a hairdresser in her own salon. Bart is a truck driver, which means he is often away from home for several days at a time. Martine has been overweight for years (BMI 27), she has been unable to lose weight.

How can you start the conversation with Martine about healthy eating during pregnancy?

Martine has indicated she would like to participate in the study. In that sense, you already have permission to talk to her about nutrition. However, things may have changed in the meantime and it is a good idea to ask for permission.

Midwife: Martine, how nice that you completed the questionnaires for the survey on empowerment and nutrition. Do you have any questions about that?

Martine: No, I think it all worked out.

Midwife: Good to hear and nice that you are participating in the study. As you already know, we are going to talk about nutrition several times. In the first conversation, I would like to look with you at what you yourself think is important, what you think is going well and what you would like to change. Do you mind if we do that now?

Martine: Yes that's fine, that's what I signed up for anyway.

Now you have outlined the framework for this conversation and have Martine's permission which makes her open to talking about nutrition. Next, you start with **step 1** of the step-by-step plan, asking how Martine views nutrition during pregnancy.

Midwife: First of all, I am curious as to why you would like to participate in this study. Can you tell me a little bit about that?

Martine: I've been trying to lose weight for a while anyway, and I'm not doing very well. I think it's also important for the baby that I eat healthy, and I hope I'll do better if we talk about that more often.

Midwife: You are absolutely right, it is indeed very important for you and your baby to eat healthy during pregnancy. Healthy eating can contribute to a happier pregnancy and delivery for yourself, and also to a healthy start for your baby. You are laying the foundation for your baby's health with this, which starts in the womb. What are some other things you consider important about food?

With this, you already have some information about Martine's motivation to eat healthier; she wants to eat healthier for the baby. Reinforce her in this motivation and then ask further what else she considers important about eating.

Martine: It should be tasty, and it shouldn't take too much time. I am often busy with my work and then it should not take me too much time.

Midwife: All very understandable. So very briefly you are concerned about the health of your baby, it should be tasty and fast. Are we so complete or are there other things you find very important?

Martine: This is true, this is the most important thing though.

You have let Martine tell you what she feels is important and summarized it. By asking if you are complete you give her the opportunity to add to it and she

feels heard and understood. It is good to have Martine now mention what she herself is happy with.

Midwife: You have already thought about your nutrition in the past time by filling out the questionnaire. Can you tell me what you yourself think is going well in your nutrition?

Martine: Well, I read once that breakfast is very important and I do that every day. A bowl of cruesli with yogurt and fresh fruit. I feel comfortable with that, because I have to walk around the salon all morning and I stand a lot. I used to often skip breakfast, then I got hungry much faster during the morning and started snacking a lot. I don't do that anymore.

You may feel the reflex here to point out that cruesli is not that healthy. Resist that tendency. Martine has made an improvement over her previous eating habits and is happy about it. Compliment her on this and let her tell you more.

Midwife: Very good to hear, Great that you picked up this much. What else is going well?

Martine: My lunch usually goes really well too, I actually always eat brown bread. Sometimes I skip lunch when the salon is very busy, but then I eat quickly in between. I also eat a lot of fruit, I like that. And I don't drink alcohol, of course I shouldn't during pregnancy, but normally I don't drink that much.

Midwife: So breakfast, lunch, fruit, no alcohol, those are quite a few things that are going well! Good to hear, you can be proud of that. When you're pregnant for the first time, new questions about food often come up. You mentioned that you think it's important to eat healthy for your baby, and I can imagine that it's not always clear what that means during pregnancy. I have a chart here with pictures of food groups. I would like to ask you to look at these and check off what you would like to talk about. You can tick more than one.

Martine ticks *"Vegetables," "Snacks/ready-to-eat,"* and *"What's better not to eat*." By letting her choose topics herself and actively tick them you increase her **commitment** to these topics.

Midwife: Ok, you want to talk about "Vegetables", "Snacks ready-to-eat" and "What better not to eat". Can you tell me a little more about these? What made you choose these groups?

Martine: Well, "vegetables" because I do know that you should eat a lot of those, but I find that difficult. "Snacks, ready-made" because I do find that tasty and easy, but I know it's not so healthy. And "what better not to eat" because I don't know that very well but that is important for the baby.

Martine here names **motives** to talk about this and she also already names a **barrier**. Ask what she wants to talk about first and come back to the barrier later (in this case: finds eating vegetables difficult).

Midwife: Do you have a preference as to which one you want to talk about first?

Martine: What I better not eat I think is most important. I have heard something about that from my girlfriends, but everyone says something different too.

Now you can give **targeted information** about what she had better not eat during pregnancy. Martine also points out that everyone says something different. Recently the dietary recommendations for pregnant women were published by the Health Council[2] which has created clarity in this area. The nutrition center has translated these recommendations into practice. In that area, refer to the website of the **Nutrition Center**. Good to also **refer to ZwangerHap**, the Nutrition Center's app on safe eating time pregnancy. Here, reliable information about food safety during pregnancy is presented very clearly and conveniently.

Once you have talked about this and checked that it is clear, you can move on to the other topics. You can make the transition by telling them that it is important for the baby's development not only to avoid the products you cannot eat, but also to eat healthy.

Midwife: You still wanted to talk about vegetables. You mentioned that you find it difficult to eat vegetables. What makes you find that difficult?

Martine: I just don't like vegetables very much and it's such a hassle to cut and prepare them. I usually eat alone in the evening because my husband is often away, and I find that too much work. Before my pregnancy I used to eat those snack tomatoes sometimes, but since I am pregnant I find them so disgusting, I don't want to think about eating a tomato.

Midwife: That's understandable, your tastes can change during pregnancy. Most of the time, fortunately, this is temporary. Eating enough vegetables is important for the baby's development, though. So can you tell me what vegetables you eat in a day now?

Martine: Well, not so much so. Yesterday evening I ate a pizza with vegetables, it had bell pepper on it. The day before I had gotten a sandwich in town for lunch, that also had vegetables on it, but I took out the tomato, and later I had some mini spring rolls, I think there were vegetables in those. But really it's not much, I know. I just don't like to cook that much.

Midwife: Is that why you checked "snacks and ready-to-eat"?

Martine: Yes. When I come home in the evening after work I'm just tired. I have been standing the whole time and then I prefer to sit on the couch and not also be in the kitchen. I'm also often the last one to close the salon, then I'm home quite late. When my husband is home, it's easier, he cooks. Wonderful if the food is ready when I come home.

Here you can magnify **ambivalence** by summarizing for yourself what Martine says with "On one side..., on the other side...". There are already motivation-enhancing aspects in this.

Midwife: Yes, I can imagine. So on the one hand you do like having something easy to eat quickly, on the other hand you indicated at the very beginning that you do know that ready-made things and snacks are not so healthy.

Martine: Yes that's right. I don't feel good about that either, and I'm already pretty heavy, but at that point I just don't know how else to do it. But I am quite afraid that I am going to gain a lot of weight because of pregnancy.

Midwife: Why are you afraid of that?

Martine: Well, it has to come off eventually and with my mother it never did. I just don't want to get any fatter.

At this stage of the conversation, a lot of information and also some barriers come up. Briefly summarize and ask her what she herself would most like to change **(step 2)**.

Midwife: On the one hand, you would like to avoid gaining a lot of weight and want to eat healthy food for the baby. It is good that you are aware that you can influence this yourself. Eating less ready-to-eat food can help limit weight gain. On the other hand, I understand that you find it difficult if your tastes have changed and that you find it a lot of work to cook if you eat alone. We have now talked about ready-to-eat, vegetables and what you really should not eat during pregnancy. The latter goes very, which is nice. You say you eat too little vegetables and too much ready-to-eat food. What would you most like to change in the coming weeks?

Martine: I don’t like eating so few vegetables. I would like to change that.

So Martine wants to eat more vegetables, but has previously indicated that she finds this difficult and also does not like it very much. On top of that, due to pregnancy, she no longer likes the cherry tomatoes she used to eat. Time to increase her motivation in **step 3**!

Midwife: If I ask you how motivated you are to eat more vegetables, where do you rank on a scale of 0-10?

Martine: I think a 5.

Midwife: What make it a 5 and not a 2?

Martine: I think eating vegetables is always good, but for the baby I'm sure it's even more important. And it also doesn't make you fat. I think that has only advantages.

You can now affirm Martine in her motivation to eat more vegetables and provide more targeted information about them. Vegetables contribute to feeling full without containing many kcal. It contains many nutrients, such as Vitamin C, vitamin A, folic acid, potassium, iron and calcium. Thus, eating vegetables helps reduce the risk of preterm birth, gestational hypertension and pre-eclampsia[8] .

Midwife: Hearing this like this, what do you think?

Martine: I really should eat more vegetables, I didn't know at all that it's so important. I really want it too, but I find it really hard right now.

Martine speaks in change language while erecting a barrier. This is a great time to move on to **step 4.**

Midwife: Now suppose you decide to eat more vegetables. What do you expect to run into then?

Martine: I think it's a combination of the fact that I don't like to cook and that I don't like a lot of vegetables. I used to eat those cherry tomatoes, but I don't have to think about that now. That makes it difficult for me.

Midwife: The cherry tomatoes you came up with are a smart solution for when you don't like to cook, though, you thought of that well. Can you think of another vegetable that you do like and is easy to eat?

Martine: I did see candy cucumbers and candy peppers the other day. I don't really like those, but I don't really hate them either. I could eat those sometime. That would be convenient and I could also take those to work.

Midwife: Good idea! If you envision this, how confident are you that it will work out on a scale of 0-10?

Martine: Well a 6. I think this should do.

Midwife: Good to hear. And what do you need to turn a 6 into an 8?

Martine: If I don't have it in the house it can be tricky, then of course I can't eat it. Actually, I just have to make sure I always have snack vegetables in the house.

Once you've discussed what might be preventing Martine from eating more snack vegetables, in **step 5** you make a concrete plan to take action.

Midwife: Good point. How are you going to make sure you do have it in the house?

Martine: I just have to put that on my grocery list by default and then take it to work.

Obstetrician: Sounds harsh! Do you always do the shopping yourself?

Martine: Often I do, but when my husband is home he does. Then I actually also have to tell him to buy me other candy vegetables.

Midwife: Yes exactly, it is very good to think about who can help you. You may write this down on this card, which you may also take home as a reminder of your goal.

Have Martine write down what is going well, what she wants to work on, and what her concrete goal is for the time ahead.

Midwife: Great that you want to eat more vegetables, you have clearly written down how you are going to approach this. You'll have your next conversation about nutrition with our dietitian, then you can review together how it went.

References

1. Timmermans YEG, Kant KD, Reijnders D, et al. Towards prepared mums (top-mums) for a healthy start, a lifestyle intervention for women with overweight and a child wish: study protocol for a randomised controlled trial in the netherlands. *Bmj open*. 2019;9(11).
2. Koletzko B, Godfrey KM, Poston L, Szajewska H, van Goudoever JB, de Waard M, et al. Nutrition During Pregnancy, Lactation and Early Childhood and its Implications for Maternal and Long-Term Child Health: The Early Nutrition Project Recommendations. Ann Nutr Metab 2019; 74(2): 93-106.
3. Garnweidner LM, Pettersen KS, Mosdøl A. Experiences with nutrition-related information during antenatal care of pregnant women of different ethnic backgrounds residing in the area of Oslo, Norway. Midwifery. 2013;29(12):e130–7.
4. Blumfield ML, Hure AJ, Macdonald-Wicks L, Smith R, Collins CE. Systematic review and meta-analysis of energy and macronutrient intakes during pregnancy in developed countries. Nutrition Reviews. 2012;70(6):322–36.
5. Vertaald na Portela A, Santarelli C. Empowerment of women, men, families and communities: true partners for improving maternal and newborn health. Br Med Bull. 2003;67(1):59–72.
6. Super S, Wagemakers A. Understanding empowerment for a healthy dietary intake during pregnancy. International journal of qualitative studies on health and well-being. 2021;16(1).
7. Super S, Beulen Y, Wagemakers A. Opportunities for dieticians to promote a healthy dietary intake in pregnant women with a low socio-economic status within antenatal care practices. Submitted.
8. Health Council of the Netherlands. Health effects of nutrient intake from supplements during pregnancy. Background document to Dietary recommendations for pregnant women. The Hague: Health Council of the Netherlands, 2021; publication no. 2021/26-A3e.
9. Klima CS, Vonderheid SC, Norr KF, Park CG. Development of the Pregnancy-related Empowerment Scale. Nursing and Health. 2015 Oct;3(5):120–7.
10. Looman M, Feskens EJM, De Rijk M, Meijboom S, Biesbroek S, Temme EHM, et al. Development and evaluation of the Dutch Healthy Diet index 2015. Public Health Nutrition. 2017;20(13):2289–99.
11. Gezondheidsraad. Voedingsaanbevelingen voor zwangere vrouwen. Den Haag: Gezondheidsraad 2021; publicatienr. 2021/26
12. Lundberg O, Peck MN. A simplified way of measuring sense of coherence: Experiences from a population survey in Sweden. Eur J Public Health. 1995;5(1):56–9.
13. Herens M. Promoting physical activity in socially vulnerable groups: a mixed method evaluation in multiple community-based physical activity programs. [Wageningen]: Wageningen University; 2016.
14. Schnittker J, Bacak V. The Increasing Predictive Validity of Self-Rated Health. PLOS ONE. 2014 Jan 22;9(1):e84933.
15. Bowling A. Just one question: If one question works, why ask several? Journal of Epidemiology & Community Health. 2005 May 1;59(5):342–5.
16. de Boer AGEM, van Lanschot JJB, Stalmeier PFM, van Sandick JW, Hulscher JBF, de Haes JCJM, et al. Is a single-item visual analogue scale as valid, reliable and responsive as multi-item scales in measuring quality of life? Qual Life Res. 2004 Mar;13(2):311–20.

Appendix 1: Conversation card

What would you like to discuss? Please indicate

You may check off more than one!

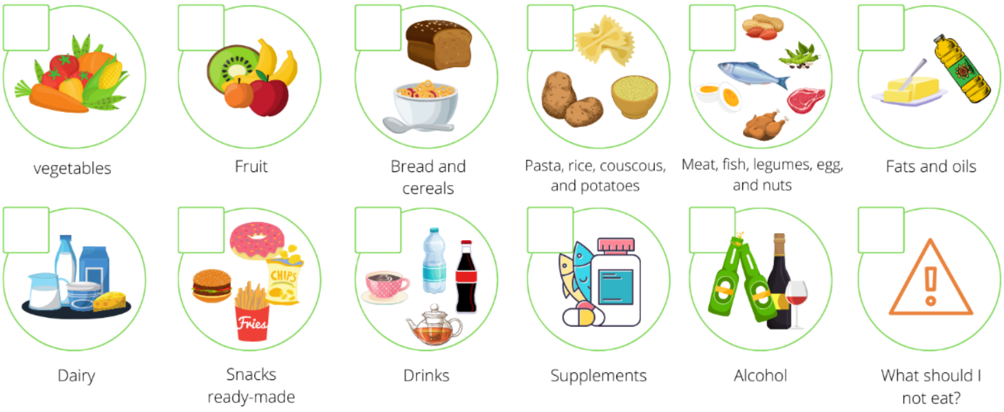

This is going well: ____________________

I want to change: ____________________

Goal for the next few weeks: ____________________

WAGENINGEN UNIVERSITY & RESEARCH

Do you want to know more?

The Nutrition Center provides information on nutrition during pregnancy on their **website**. *www.voedingscentrum.nl/zwanger*

On the Nutrition Center's **YouTube** channel, you can find videos on such topics as: Nutrition during pregnancy and breastfeeding | Safe eating | The Wheel of Five | Recipes

YouTube

Do you ever doubt what you **can** and **cannot safely** eat when you are pregnant? Download the app ZwangerHap. You will find clear and reliable information.

ZwangerHap

Notes

____

____

____

____

____

____

____

POWER4 GEZOND ZWANGER

WAGENINGEN UNIVERSITY & RESEARCH
