# Supplementary material for: Pregnant Women's Experiences With a Collaborative Midwife‐Dietitian Empowerment Programme to Improve Diet Quality
Source: J Hum Nutr Diet. 2025 Feb 11;38(1):e70027. doi: 10.1111/jhn.70027 (PMC11813828; doi:10.1111/jhn.70027)
Supplement: Supplementary file 2 — Supporting information. [file JHN-38-0-s001.pdf]

Supplementary file 2: Interview guide

Van Lonkhuijzen, R. M., Prins, S., van Loghem, F., De Vries, J. H. M., & Wagemakers, A. (2024). Pregnant women's experiences with an empowerment programme to improve diet quality.

1. Warm-up questions:
    a. How are you doing?
    b. What is your family composition?
    c. How did you experience your pregnancy?
2. Introducing timelining-tool
3. General information pregnancy-timeline
    a. How did you experience the care during your pregnancy?
    b. How did you experience the care regarding nutrition?
    What were important moments for you in the beginning of your pregnancy? Can you remember any important moments thereafter?
4. General information P4HP
    You signed up for P4HP at the beginning of your pregnancy. Therefore, you experienced four additional moments during your pregnancy with your midwife and a dietitian to talk about nutrition.
    a. What was the reason you participated in P4HP? *(motivation)*
    b. What do you remember from the consultations?
    ➔ **Questioning consultations 1, 2, 3 and 4 separately**
    c. How did you experience consultation x? (x= consult 1, 2, 3, 4)
        i. Can you tell us how these consultations went?
        ii. Can you remember any important moments from these consultations?
        iii. What did you learn from these consultations?
    d. If you had to give P4HP a grade, what grade from 1 to 10 would it be? Why a ... and not a ...? What needs to happen to make this grade a 9 or 10?
5. Context diet quality
    We are also specifically curious about what happened you regarding your diet.
    1. What does a healthy diet entail for you during pregnancy? Has this changed from before pregnancy?
        i. How has your diet evolved during pregnancy?
        ii. What has changed? Did you start eating certain food products or not? (risky/health promoting food products)

iii. Can you remember specific trigger points when your eating habits changed during your pregnancy?
iv. Did the discussions with the midwife/dietician influence this? Did other factors contribute to this?
v. Did you find it easy/difficult to stick to this pattern? (strategies/challenges)
vi. Would you handle food differently in a future pregnancy? If so, how? (motivation)
vii. Are there any important lessons you learned about yourself and healthy eating during this pregnancy?

6. Context empowerment

   P4HP aimed to focus on pregnant woman's desires and abilities to eat healthier during nutrition consultations, and that the woman has control during these conversations.

   a. Did you experience this? (open dialogue)
   b. Did you consider healthy eating during pregnancy to be your own responsibility? (control)
      i. Do you feel that you have control over what you eat during your pregnancy? How? (control)
      ii. Does this affect your diet? How?
      iii. How do you assess your own knowledge regarding healthy eating? (control)
      iv. Did you feel heard during conversations with the midwife/dietitian? Were all your questions answered? (control/ open dialogue)
      v. Did you receive tailored advice from your midwife and the dietician? (Personalised information)
      vi. Were things discussed during the consultation that you found difficult? (different motivations)
   2. What was the influence of your environment on your eating behavior? (social environment)
      i. Was this discussed during the consultation? (open dialogue)

7. Closing
   a. Is there anything we haven't discussed yet that you would like to tell/discuss?

8. Thanking participants for their participation
